# Antimicrobial Peptides and Biomarkers Induced by Ultraviolet Irradiation Have the Potential to Reduce Endodontic Inflammation and Facilitate Tissue Healing

**DOI:** 10.3390/pharmaceutics14091979

**Published:** 2022-09-19

**Authors:** Kimberly A. Morio, Robert H. Sternowski, Erliang Zeng, Kim A. Brogden

**Affiliations:** 1 Apex Endodontics, Hiawatha, IA 52233, USA; 2 Softronics, Ltd., Marion, IA 52302, USA; 3 Division of Biostatistics and Computational Biology, College of Dentistry, The University of Iowa, Iowa City, IA 52242, USA; 4 College of Dentistry, The University of Iowa, Iowa City, IA 52242, USA

**Keywords:** ultraviolet irradiation, UV, UVC, UVB, UVA, antimicrobial peptides, chemokines, cytokines, endodontic, inflammation, pain, tissue healing

## Abstract

Background: Ultraviolet (UV) irradiation can modulate host immune responses and this approach is a novel application for treating endodontic infections and inflammation in root canals. Methods: A dataset of UV-induced molecules was compiled from a literature search. A subset of this dataset was used to calculate expression log2 ratios of endodontic tissue molecules from HEPM cells and gingival fibroblasts after 255, 405, and 255/405 nm UV irradiation. Both datasets were analyzed using ingenuity pathway analysis (IPA, Qiagen, Germantown, MD, USA). Statistical significance was calculated using Fisher’s exact test and z-scores were calculated for IPA comparison analysis. Results: The dataset of 32 UV-induced molecules contained 9 antimicrobial peptides, 10 cytokines, 6 growth factors, 3 enzymes, 2 transmembrane receptors, and 2 transcription regulators. These molecules were in the IPA canonical pathway annotations for the wound healing signaling pathway (9/32, *p* = 3.22 × 10^−11^) and communication between immune cells (6/32, *p* = 8.74 × 10^−11^). In the IPA disease and function annotations, the 32 molecules were associated with an antimicrobial response, cell-to-cell signaling and interaction, cellular movement, hematological system development and function, immune cell trafficking, and inflammatory response. In IPA comparison analysis of the 13 molecules, the predicted activation or inhibition of pathways depended upon the cell type exposed, the wavelength of the UV irradiation used, and the time after exposure. Conclusions: UV irradiation activates and inhibits cellular pathways and immune functions. These results suggested that UV irradiation can activate innate and adaptive immune responses, which may supplement endodontic procedures to reduce infection, inflammation, and pain and assist tissues to heal.

## 1. Introduction

Physical traumas, factures, erosions, and local infections, including caries and periodontal disease, on human teeth are among the conditions that lead to endodontic disease [1]. These conditions often create ‘barrier defects’ that allow entry of opportunistic oral microbiota into the underlying dental pulp tissue. Invading microorganisms can then develop into polymicrobial biofilms containing *Archaebacteria*, *Eubacteria*, yeast, and fungal species [2,3,4]. The resulting infections can induce inflammation and pain in the root canal systems. The associated craniofacial pain can be severe and significantly impact the patient’s quality of life and day-to-day comfort.

Current standard-of-care treatment for endodontic emergencies and treatment include opening the tooth; exposing underlying inflammatory tissue or infection; and creating an open instrumented root canal to the tooth apex, removing the inflammatory tissue, canal exudate, necrotic tissue, and tissue debris [3]. The root canal is then irrigated with sodium hypochlorite (NaOCl) to cleanse and dissolve the canal of the remaining tissue and debris, followed by the irrigation of ethylenediaminetetraacetic acid (EDTA) to remove the smear layer and open the dentinal tubules. The root canal is completed by sealing the canal space with endodontic sealer and gutta percha. The access opening is filled with amalgam or a composite material, and in some cases, a crown is advocated to enhance tooth integrity and adequate seal.

Unfortunately, the standard-of-care treatment is unable to completely clean the canal space and residual pulp tissue debris, missed areas of infection, and remnant microorganisms in the dentin tubules along the sides of the root canal can be left; resulting in potential persist reinfections and apical chronic inflammation [3]. Irrigation with NaOCl is the gold standard, but extrusion of this irrigant apically can be extremely detrimental to the apical tissues. The damaged apical tissues by the NaOCl have little chance to regenerate and heal with no opportunities for activation of local innate or adaptive immune responses in the area.

An emerging and novel concept is to treat instrumented root canals with ultraviolet (UV) irradiation during the standard-of-care treatment procedure [5,6]. UVC irradiation kills microorganisms isolated from endodontic infections [6,7,8]. Brief treatment with UV irradiation would also modulate host immune responses [9,10]. UV irradiation induces an influx of cells and the production of antimicrobial peptides (AMPs); chemokines, cytokines, and biomarkers (CCBMs); and other molecules that alter lesion pathogenesis and facilitates local tissue healing [9,10]. UVC induces the secretion of CCBMs in HEPM cells and gingival fibroblasts related to endodontic tissue regeneration [6].

**Figure 1:**
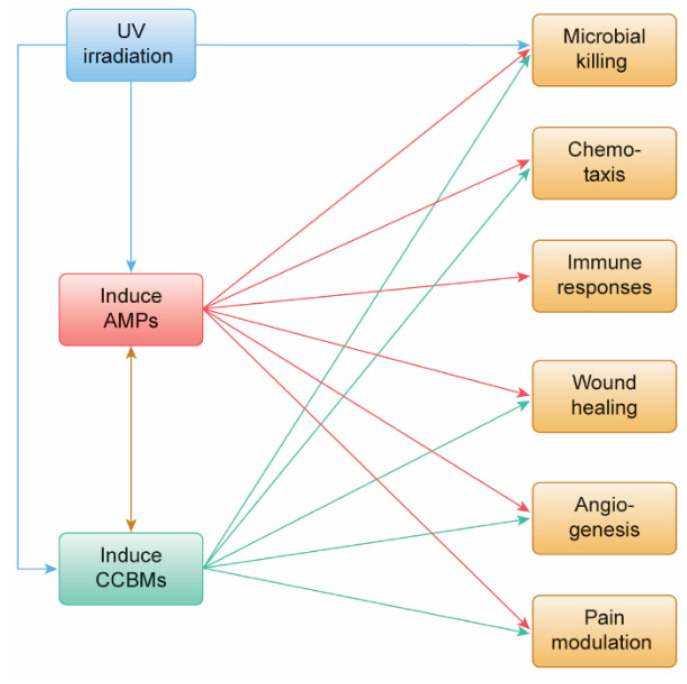
A schematic diagram of the proposed effects of UV irradiation on endodontic infection and inflammation, pain, and tissue healing. UV irradiation can kill microorganisms directly (blue line). UV irradiation can also induce host cells to express antimicrobial peptides (AMPs) and chemokines, cytokines, and biomarkers (CCBMs) (blue lines). AMPs can kill microorganisms (red line); induce the production of CCBMs (brown line); and induce chemotaxis, modulate immune responses, assist in wound healing, play a role in angiogenesis, and reduce pain (red lines). CCBMs can kill microorganisms (green line); induce the production of AMPs (brown line); and induce chemotaxis, modulate immune responses, assist in wound healing, play a role in angiogenesis, and reduce pain (green lines).

In this study, we were interested in identifying molecules expressed or secreted from cells and tissues after UV irradiation. We first searched the PubMed literature to identify AMPs or CCBMs reported to have up- or down-levels of mRNA expression or secretion in response to UV irradiation treatment. We used a bioinformatics approach with ingenuity pathway analysis (IPA, Qiagen, Germantown, MD, USA) to associate their expression with specific innate and adaptive immune responses. We then used the concentrations of 13 CCBMs in tissue culture media of HEPM cells and gingival fibroblasts after treatment with 255, 405, or 255/405 UV irradiation [6] as (i) wet lab experimental data to validate claims from the bioinformatics data, and (ii) to assess the ability of UV irradiation to activate or inhibit cellular pathways related to innate and adaptive immune responses. We hypothesized that UV irradiation would induce host cells and tissues to express AMPs and CCBMs (Figure 1) and these molecules would be important in future treatments designed to reduce endodontic infection and inflammation, modulate endodontic pain, and assist in endodontic tissue healing.

## 2. Materials and Methods

### 2.1. Dataset of UV-Induced Molecules

We searched the PubMed literature using UVC, UVB, UVA, chemokines, cytokines, and antimicrobial peptides as search terms linked in various combinations, using Boolean operators to identify AMPs or CCBMs reported to have up- or down-levels of mRNA expression or secretion in response to UV irradiation. AMPs and CCBMs were combined into a single dataset (Table 1).

### 2.2. Subset of Endodontic Tissue Molecules

**Figure 2:**
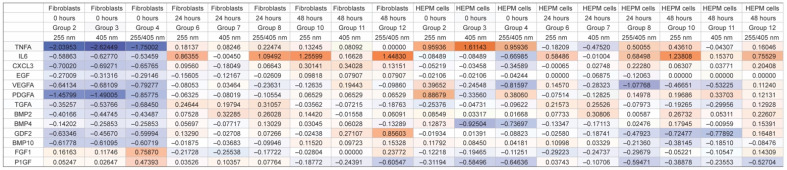
Expression Log2 ratios of 13 chemokine, cytokine, and biomarker (CCBMs) concentrations reported in tissue culture media of HEPM cells and gingival fibroblasts at 0, 24, and 48 h after treatment with 255 nm, 405 nm, or 255/405 nm UV irradiation. Expression was calculated as the log2 ratio of the mean of each treatment after UV irradiation over the mean of the untreated control for that same cell type, UV irradiation wavelength, and time period. Groups are shown as a heatmap, where blue represents inhibition (negative values), white represents midpoint, and orange represents activation (positive values).

Morio et al. reported the concentrations of 13 CCBMs in tissue culture media of HEPM cells and gingival fibroblasts at 0, 24, and 48 h after treatment with 255, 405, or 255/405 UV irradiation [6]. We used these CCBMs as (i) wet lab experimental data to validate claims from the bioinformatics data, and (ii) to assess the ability of UV irradiation to activate or inhibit cellular pathways related to immune functions. For this, levels of CCBM expression were calculated as the expression log2 ratios of the mean of each treatment after UV irradiation, divided by the mean of the respective control for the same treatment (Figure 2).

### 2.3. Analysis

We used ingenuity pathway analysis (IPA, Qiagen, Germantown, MD, USA) to assess whether the molecules in this study were related to the activation of innate and immune mechanisms. Two types of analysis were performed.

IPA core analysis was run on the list of 32 molecules from the literature dataset in Table 1 and used to assess whether the IPA canonical pathway and IPA diseases and function annotations were predicted to be associated with relevant diseases, immune pathways, and immune functions. Statistical significance was calculated using Fisher’s exact test and significant *p* values (*p* < 0.05) were reported.

IPA comparison analysis was run on the expression log2 ratios of the 13 molecules and used to assess whether the IPA canonical pathway and IPA diseases and function annotations were predicted to be activated or inhibited after UV irradiation. Statistical differences were determined using activation z-scores calculated from the mean of each treatment expression log2 ratio. The activation z-score was determined by IPA as reported by Kramer et al. [11] and makes predictions based on the direction of gene activation or inhibition.

IPA comparison analysis was also used to assess whether the IPA canonical pathway predicted any effects of the activated or inhibited 13 molecules on downstream regulation of gene expression for other innate or adaptive immune functions.

## 3. Results

### 3.1. Dataset of 32 UV-Induced Molecules

We identified 32 unique molecules reported to be expressed after UV irradiation of cells and tissues (Table 1). There were 9 antimicrobial peptides (Supplementary Table S1), 10 cytokines, 6 growth factors, 3 enzymes, 2 transmembrane receptors, and 2 transcription regulators (Supplementary Table S2).

IPA analysis predicted that UV irradiation can induce molecules that are involved in innate and adaptive immune responses. IPA canonical pathway annotations of these 32 molecules (Table 1) were predicted to be associated with cellular stress and injury. This category included the wound healing signaling pathway (9/32, *p* = 3.22 × 10^−11^). Annotations were also associated with cellular immune responses, and this category included the role of cytokines in mediating communication between immune cells (6/32, *p* = 8.74 × 10^−11^), communication between innate and adaptive immune cells (5/32, *p* = 7.44 × 10^−3^), the Th1 and Th2 activation pathway (4/32, *p* = 8.01 × 10^−5^), the Th1 pathway (3/32, *p* = 5.81 × 10^−4^), and the Th2 pathway (3/32, *p*= 8.13 × 10^−4^). The top relevant pathway annotations are listed in Table 2 and all the relevant annotations ranked by their −log(*p* value) are listed in Supplementary Table S3. This list contains relevant annotations within categories on cytokine signaling; growth factor signaling; intracellular and second messenger signaling; cellular growth, proliferation, and development; cellular immune response; and organismal growth and development.

IPA disease and function annotations of these 32 molecules (Table 1) were predicted to be associated with innate and adaptive immune responses, applicable to reducing infection and inflammation and assisting in tissue healing. These included antimicrobial response, inflammatory response, cell-to-cell signaling and interaction, cellular movement, immune cell trafficking, cell death and survival, cellular growth and proliferation, cellular development, hematological system development and function, hematopoiesis, lymphoid tissue structure and development, tissue development, and tissue morphology. Examples of these functions are listed in Table 2 and all the relevant IPA disease and function annotations are listed in Supplementary Table S4.

### 3.2. Subset of 13 Endodontic Tissue Molecules

**Figure 3:**
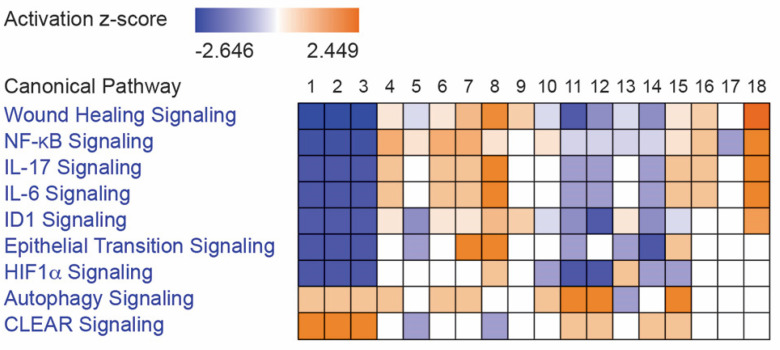
IPA comparison analysis of 18 observations from fibroblasts (observations 1–9) and HEPM cells (observations 10–18) at 0 h (observations 1–3, 10–12), 24 h (observations 4–6, 13–15), and 48 h (observations 7–9, 16–18) after treatment with 255 nm (observations 1, 4, 7, 10, 13, and 16), 405 nm (observations 2, 5, 8, 11, 14, 17), or 255/405 nm (observations 3, 6, 9, 12, 15, and 18) irradiation. Groups are shown as a heatmap, where blue represents inhibition (negative values), white represents midpoint, and orange represents activation (positive values). Numerous IPA canonical pathways were inhibited shortly after irradiation (0 h) but activated at 24 and 48 h. Fibroblasts and HEPM cells both were strongly activated by 405 nm and 255/405 nm UV irradiation treatments.

IPA analysis also predicted that different wavelengths of UV irradiation might selectively regulate gene expression for innate or adaptive immune functions. IPA comparison analysis of the log2 ratios of the concentrations of 13 CCBMs in tissue culture media of HEPM cells and gingival fibroblasts at 0, 24, and 48 h after treatment with 255, 405, or 255/405 UV irradiation identified differences in expression across 18 observations, representing 2 cell lines, 3 UV treatments, and 3 time periods (Figure 3). The expression log2 ratios of the 13 CCBMs varied from −2.6245 to 1.6114. Relevant IPA canonical pathways in Figure 3 were related to cellular stress and injury (wound healing signaling pathway, the CLEAR signaling pathway, HIF1α signaling pathway, and autophagy), cytokine signaling (IL17 signaling, IL6 signaling, and NF-κB signaling), growth factor signaling (regulation of the epithelial mesenchymal transition by growth factors pathway), and organismal growth and development (ID1 signaling pathway). IPA comparison analysis predicted that UV irradiation activated or inhibited pathways depending upon the cell type, wavelength of treatment, and time after treatment. Numerous IPA canonical pathway annotations were inhibited shortly after irradiation (0 h) but activated at 24 and 48 h. Fibroblasts and HEPM cells both were strongly activated by 405 nm and 255/405 nm UV irradiation treatments (Figure 3).

**Figure 4:**
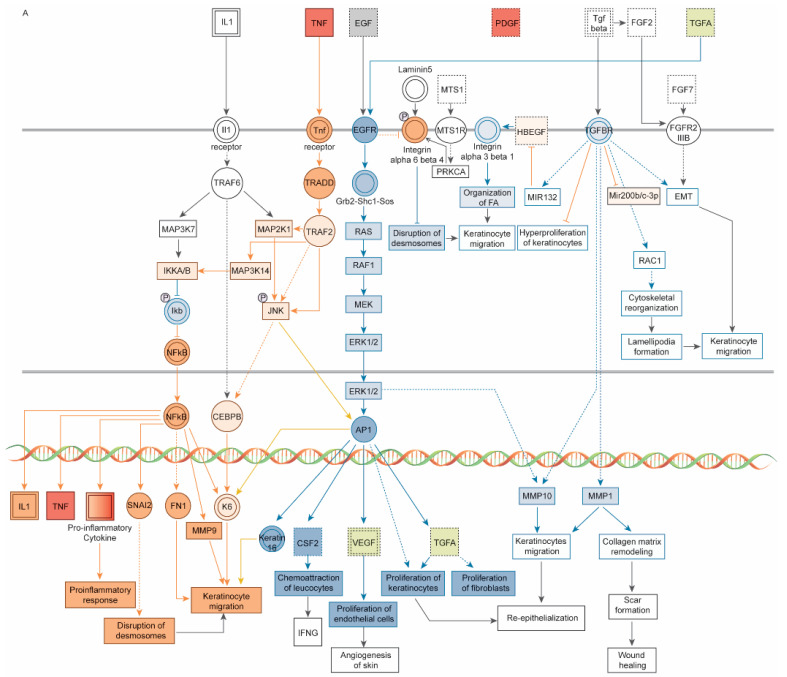

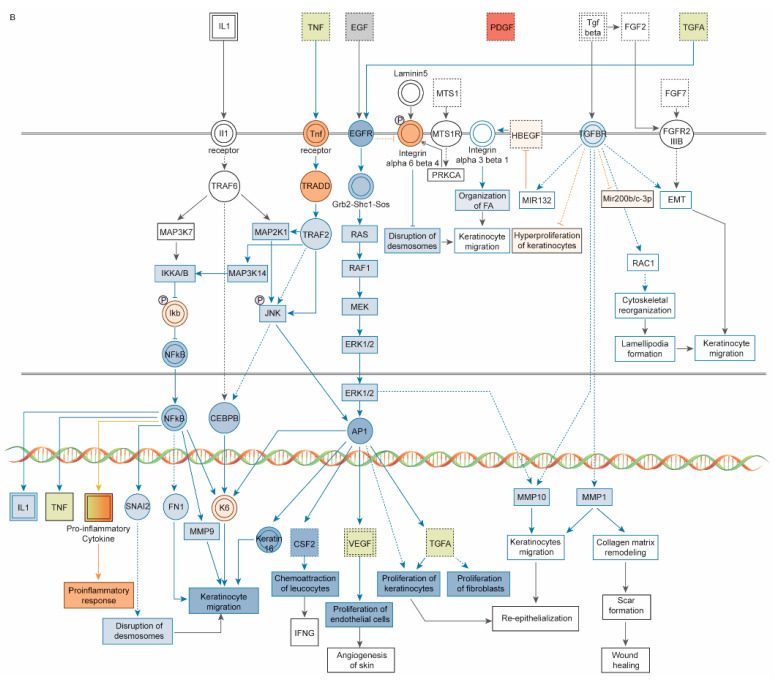

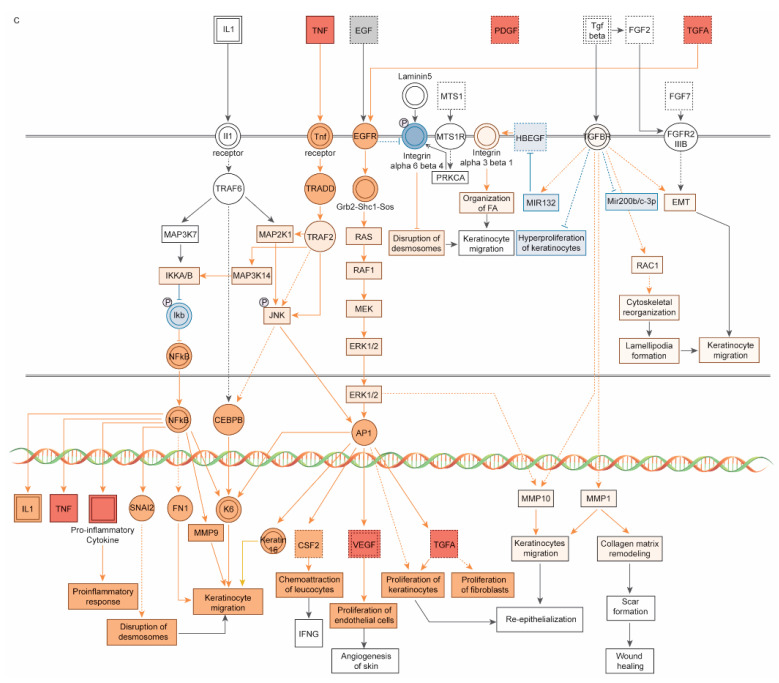
Schematic diagrams of the wound healing signaling pathway, prepared using Ingenuity Pathway Analysis software (IPA, Qiagen, Germantown, MD), showing both inhibited and activated pathway signaling in HEPM cells 48 h after (**A**) 255 nm irradiation, (**B**) 405 nm irradiation, and (**C**) 255/405 nm irradiation. Signaling starts via TNF binding to the TNF receptor; EGF and TGFA binding to the EGFR; and TGFB binding to the TGFBR. These pathways signal through TRADD and TRAF2 to JNK and through RAS, RAF, and MEK to ERK1/2. Signaling continues to NF-κB, CEBPB, and AP-1 to activate additional CCBMs, leading to proinflammatory responses, disruption of desmosomes, chemoattraction of leukocytes, migration and proliferation of fibroblasts and cells, collagen matrix remodeling, and wound healing pathways. Pathway molecules in red indicate activation and molecules in green indicated inhibition. Signaling connections in orange indicate pathway activation and signaling connections in blue indicate pathway inhibition.

Within the wound healing signaling pathway, for example, after 0 h of 255, 405, and 255/405 nm irradiation, fibroblasts and HEPM cells (observations 1–3, 10–12) had negative z-scores, predicting inhibition of pathway signaling, whereas after 24 and 48 h after 255, 405, and 255/405 nm irradiation, fibroblasts (observations 4–9) and after 48 h of 255, 405, and 255/405 nm irradiation, HEPM cells (observations 16–18) had positive z-scores, predicting activation of pathway signaling. There were also conditions predicted to activate some pathways yet inhibit others (observations 3, 9, 12, 15, and 18). Here, treatment with cells with 255/405 nm irradiation contained both inhibited and activated pathway signaling. After 48 h of 255/405 nm irradiation, HEPM cells (observation 18) had a positive z-score but contained both inhibited and activated pathway signaling (Figure 4A–C). Signaling was predicted to occur through binding of molecules to the TNF receptor, EGFR, and TGFBR and signaling through JNK and ERK1/2 to transcription factors NF-κB, CEBPB, and AP-1. This was predicted to activate additional CCBMs, leading to proinflammatory responses, disruption of desmosomes, chemoattraction of leukocytes, migration and proliferation of fibroblasts and cells, collagen matrix remodeling, and wound healing pathways.

### 3.3. Modulating Endodontic Pain

While UV irradiation is not known to be directly anti-nociceptive, it does act on ERK, p38 MAPK, JNK, and NF-κB signaling pathways (Supplementary Figure S1) that produce AMPs and CCBMs that are involved in pain reduction (Supplementary Figure S2). Many of the molecules induced by UV irradiation (Table 1) can be pro-nociceptive, nociceptive, and anti-nociceptive stimuli. AMPs can be anti-nociceptive stimuli or pro-nociceptive stimuli, chemokines can be pro-nociceptive or nociceptive stimuli, and growth factors can be anti-nociceptive stimuli.

## 4. Discussion

We identified a list of 32 molecules expressed or secreted from cells and tissues after UV irradiation and used a bioinformatics approach to show that they were related to wound healing and innate and adaptive immune functions, including chemotaxis, movement, growth, and proliferation of cells. We then used a subset of 13 osteoinductive, angiogenic, proliferative, and proinflammatory molecules to show that HEPM cells and gingival fibroblasts treated with 255, 405, and 255/405 nm UV irradiation had different expression profiles. These results suggested that UV irradiation can activate innate and adaptive immune responses, which may supplement endodontic procedures to reduce infection, inflammation, and pain and assist tissues to heal.

Wound healing is a complex process, whereby secreted molecules from infected, injured, or damaged cells and tissues attract a variety of inflammatory cells to the injured site (inflammatory phase). Once present, inflammatory cells release additional CCBMs. These include growth factors that attract and transform fibroblasts and molecules that stimulate attracted cells to proliferate and stimulate other cells to begin forming new capillaries and blood vessels (proliferative phase). Tissue development, angiogenesis, and vasculogenesis occurs (maturation and remodeling phase). IPA identified 13/32 molecules (*p* = 1.53 × 10^−20^) in the IPA canonical pathway annotations associated with these phases in the wound healing signaling pathway (Table 2, Supplementary Table S3). Of the 13 molecules, TNFA, a proinflammatory cytokine, is secreted by activated monocytes, macrophages, B-cells, T-cells, and fibroblasts; IL6 regulates immune and inflammatory responses, including B-cell differentiation and antibody production; and IL10 inhibits the expression of pro-inflammatory cytokines, but enhances humoral immune responses and attenuates cell mediated immune reactions. CSF2 stimulates the development of neutrophils and macrophages. Growth factors including FGF1, FGF2, TGFA, TGFB1, and VEGFA are involved in cell motility; cell proliferation; cell growth and differentiation; redistribution of tissue; angiogenesis and vascular permeability of endothelial cells; and synthesis and deposition of the extracellular matrix. FN1 and collagen from fibroblasts allow tissues to contract [12], SMAD3 and SMAD4 play roles in the signaling of TGFB1 [13], and ICAM1 provides adhesion between endothelial cells and leukocytes after stress or injury.

Innate immunity is a type of nonspecific host resistance without memory, involving soluble molecules and cells [14]. Stimulation of receptors activates several cellular pathways, resulting in the production of AMPs and inflammatory cytokines. Stimulation also leads to changes in cellular metabolism, upregulation of numerous genes involved in cell defense and pathogen restriction, and the induction of regulated cell death [14].

AMPs are a large component of innate immune responses and UV irradiation induces their transcription and secretion (Supplementary Table S1). Their ability to modulate both innate immune responses, cellular immunity, and angiogenesis are very well known [15,16,17,18]. IPA identified 14/32 molecules involved in antimicrobial and antibacterial response annotations (Supplementary Table S4). These included CAMP (LL37), the defensin family (DEFB1, DEFB103A/DEFB103B, and DEFB4A/DEFB4B), the S100 family of calcium binding proteins (S100A7, S100A8, S100A9, and S100A12), and RNASE7. It also included the chemokine CCL20, which has antimicrobial activity [19] and cytokines IL6, IL10, TNFA, and TGFB1, which contribute to the antibacterial response. So far, AMP expression and secretion has been reported to occur in a narrow range from 280 to 400 nm (Supplementary Table S1). AMP expression is increased after 280–313 nm irradiation [20,21], but not after 340 to 400 nm irradiation [22,23]. Thus, UV irradiation of an infected root canal would not have to kill 100% of microorganisms, but simply reduce the microbial infection burden to a level that could be managed by UV-induced innate immune mechanisms.

CCBMs are large components of innate immune responses and UV irradiation induces their transcription and secretion (Supplementary Table S2), CCBM expression and secretion has been reported to occur in a wider range from 254 to 404 nm (Supplementary Table S2). In irradiated cells, CCBM expression is increased for many CCBMs after 200 to 320 nm irradiation but decreased for others, such as BMP10 and FGF1 in HEPM cells and VEGF in fibroblasts [6]. At 340 to 405 nm irradiation, cells had increased levels of IL6, CXCL8, and CSF mRNA expression and secretion in keratinocytes [24,25] but decreased levels of secreted FGF1 in fibroblasts [6]. In irradiated human and murine skin at 2–3 MED (minimal erythemal dose), there were increased levels of immunostaining for CCL2, CCL20, CXCL1, CXCL8, ICAM1, IL1, IL10, SELE, SMAD3, SMAD4, TGFA, TGFB, TNFA, and VEGF 24–48 h after exposure [26,27,28].

UV-induced AMPs and CCBMs would have a variety of common functions (Figure 1). In addition to their potent antimicrobial activity mentioned above, these UV-induced molecules can chemoattract a variety of cells important to both immune protection and wound healing. Defensins attract keratinocytes, dendritic cells, and T-cells [29,30] and CAMP (LL37) attracts fibroblasts, microvascular endothelial cells, and human umbilical vein endothelial cells [31]. AMPs can regulate proinflammatory CCBM production [32]. At lower concentrations, defensins do not induce TNFA or IL1B expression in monocytes or macrophages [33,34]. However, at higher concentrations, defensins induce CCBM production in epithelial cells, keratinocytes, monocytes, and macrophages [30,35,36] and CAMP (LL37) induces CXCL8 in epithelial cells and macrophages [37]. Finally, AMPs and CCBMs play a direct role in wound healing, angiogenesis, and vasculogenesis [18]. DEFB4A (HBD2) increases keratinocyte proliferation [30,38] and CAMP (LL37) increases fibroblast proliferation, induces human microvascular endothelial cell and human umbilical vein endothelial cell proliferation and stimulates re-epithelialization [31,39,40].

UV irradiation also suppresses cellular immunity and acts primarily on T-cell-mediated immune reactions [41,42,43]. This application has been used to treat several T-cell-mediated diseases, including graft-versus-host disease and systemic scleroderma [44]. UV irradiation alters antigen specificity, alters antigen-presenting cell function, acts on effector and regulatory T-cells [41] and induces the production of CCBMs [42]. For example, UV irradiated dendritic cells do not present antigens effectively, and thus induce regulatory T-cells (CD4^+^CD25^+^), but not effector T-cells [41]. UV irradiation can lead to T-cell tolerance and prevents the priming of antigen-specific CD8^+^ T-cells (in models of contact hypersensitivity) independent of conventional CD4^+^ regulatory T-cells [44]. Tolerant CD8^+^ T-cells prevented migration of dendritic cells and prevented priming of other CD8^+^ T-cells. TGFB and immunosuppressive IL10 are regulatory T-cell-associated cytokines [10,45].

There are differences based on the specific wavelength. UVB induces the infiltration of immature inflammatory myeloid CD11c^+^ bDCA1^-^ dendritic cells, which may have a suppressive function [10]. UVA1 does not induce IL10, but does suppress the production of TNFA and IL12, and contributes to cis-UCA isomerization [10,46]. Immune suppression may be dependent upon the extent of UV irradiation-induced damage to DNA [41]. However, to what extent the secondary immunostimulatory effects of UV-induced AMPs and CCBMs offset the immunosuppressive effects of immune cells is not yet known.

Many oral related infections, inflammation, and tissue injury/peripheral nerve injury can be stimuli that activate receptors on the surface of cells, initiating signal transduction through MAPK and NF-κB signaling pathways (Supplementary Figure S2) [47]. The MAPK pathway regulates proinflammatory and pronociceptive molecules involved in inflammation and pain [48]. Nociceptive activity or nerve injury stimuli signal through raf and MEK1/2 to ERK in the cytoplasm and transcription factor CREB in the nucleus [48]. Chemokines (FKN), cytokines (TNFA), and nerve injury stimuli signal through TAK1 and MKK3,6 to p38 MAPK in the cytoplasm and on to transcription factor ATF-2 in the nucleus [48]. Cytokines (TNFA), growth factors BFGF (FGF2), and nerve injury stimuli signal through MLK3/MEKK1 and MKK4,7 to JNK in the cytoplasm and on to transcription factor c-Jun (AP-1) in the nucleus [48].

UV irradiation also alters the expression of ERK, p38 MAPK, JNK, and NF-κB signaling pathways [49] (Supplementary Figure S1) and it is possible that treatment of endodontic infections, inflammation, and tissue injury/peripheral nerve injury can modulate downstream production of CCBMs and be a potential intervention at these nodes to mitigate acute or chronic pain. Phosphorylation of ERK in nerve injury is induced early, is long lasting, and is involved in the induction of pain (Supplementary Figure S2). Suppressing this step in ERK is thought to be a promising strategy for treatment of neuropathic pain [50]. Likewise, targeting the p38 MAPK pathway and its signaling is also thought to be a potential therapeutic strategy for pain management [51].

Our analysis and results support those of Ou and Peterson [9] and Vieyra-Garcia et al. [10] and also suggest that UV irradiation can induce the production of AMPs and CCBMs. Our results also suggest that the production of these molecules can induce the innate and adaptive immune responses involved in attenuating infection, inflammation, and pain and enhancing healing and regeneration of tissue. However, these results are based on bioinformatics analysis of molecules induced by UV irradiation reported in the literature and produced in culture from cells treated with UV irradiation. These concepts and results form a strong hypothesis for future studies and should be examined in detail.

## 5. Conclusions

In summary, UV irradiation has the ability to kill microorganisms, but could also be used to activate innate and adaptive immune mechanisms in endodontic root canals directly or through UV-induced molecules. UV irradiation-induced effects appear to be wavelength specific and could supplement procedures to reduce infection, to reduce inflammation, and to facilitate local tissue healing.

## Figures and Tables

**Table 1:** Antimicrobial peptides (AMPs); chemokines, cytokines, and biomarkers (CCBMs); and other mediators were identified from ingenuity pathway analysis (IPA, Qiagen, Germantown, MD, USA), combined into a single dataset below, annotated for their IPA symbol, Entrez Gene name, Entrez Gene ID (human), cellular location, and function type.

Symbol	Entrez Gene Name	Entrez Gene ID (Human)	Location	Function Type
BMP10	Bone morphogenetic protein 10	27302	Extracellular space	Growth factor
CAMP	Cathelicidin antimicrobial peptide	820	Cytoplasm	Other
CCL2	C-C motif chemokine ligand 2	6347	Extracellular space	Cytokine
CCL20	C-C motif chemokine ligand 20	6364	Extracellular space	Cytokine
CSF2	Colony stimulating factor 2	1437	Extracellular space	Cytokine
CXCL1	C-X-C motif chemokine ligand 1	2919	Extracellular space	Cytokine
CXCL2	C-X-C motif chemokine ligand 2	2920	Extracellular space	Cytokine
CXCL3	C-X-C motif chemokine ligand 3	2921	Extracellular space	Cytokine
CXCL8	C-X-C motif chemokine ligand 8	3576	Extracellular space	Cytokine
DEFB1	Defensin beta 1	1672	Extracellular space	Other
DEFB103B	Defensin beta 103B	55894	Extracellular space	Other
DEFB4A	Defensin beta 4A	1673	Extracellular space	Other
FGF1	Fibroblast growth factor 1	2246	Extracellular space	Growth factor
FGF2	Fibroblast growth factor 2	2247	Extracellular space	Growth factor
FN1	Fibronectin 1	2335	Extracellular space	Enzyme
ICAM1	Intercellular adhesion molecule 1	3383	Plasma membrane	Transmembrane receptor
IL6	Interleukin 6	3569	Extracellular space	Cytokine
IL10	Interleukin 10	3586	Extracellular space	Cytokine
PI3	Peptidase inhibitor 3	5266	Extracellular space	Other
PIGF	Phosphatidylinositol glycan anchor biosynthesis class F	5281	Cytoplasm	Enzyme
RNASE7	Ribonuclease A family member 7	84659	Extracellular space	Enzyme
S100A7	S100 calcium binding protein A7	6278	Cytoplasm	Other
S100A8	S100 calcium binding protein A8	6279	Cytoplasm	Other
S100A9	S100 calcium binding protein A9	6280	Cytoplasm	Other
S100A12	S100 calcium binding protein A12	6283	Cytoplasm	Other
SELE	Selectin E	6401	Plasma membrane	Transmembrane receptor
SMAD3	SMAD family member 3	4088	Nucleus	Transcription regulator
SMAD4	SMAD family member 4	4089	Nucleus	Transcription regulator
TGFA	Transforming growth factor alpha	7039	Extracellular space	Growth factor
TGFB1	Transforming growth factor beta 1	7040	Extracellular space	Growth factor
TNF	Tumor necrosis factor	7124	Extracellular space	Cytokine
VEGFA	Vascular endothelial growth factor A	7422	Extracellular space	Growth factor

**Table 2:** Ingenuity pathway analysis (IPA, Qiagen, Germantown, MD, USA) was used to assess whether the biomarkers in the literature dataset (*n* = 32) would participate in the activation of innate and immune mechanisms applicable to reducing endodontic infection, reducing inflammation, and assisting in endodontic tissue healing. Representative IPA canonical pathways annotations were associated with cellular stress and injury; cytokine signaling; cellular immune response. Representative IPA diseases or functions annotations were associated with antimicrobial response, cell-to-cell signaling and interaction, cellular movement, hematological system development and function, immune cell trafficking, and inflammatory response.

IPA Function	*p*-Value	No.	Identification of Molecules
**Canonical Pathway Annotations**			
**Cellular Stress and Injury**			
Wound healing signaling pathway	3.22 × 10^−11^	9	CSF2, CXCL8, FGF2, FN1, IL6, TGFA, TGFB1, TNF, VEGFA
**Cytokine Signaling**			
IL17 signaling	8.43 × 10^−20^	13	CCL2, CCL20, CSF2, CXCL1, CXCL3, CXCL8, DEFB1, DEFB103A/DEFB103B, DEFB4A/DEFB4B, IL6, TGFB1, TNF, VEGFA
IL6 signaling	2.53 × 10^−5^	4	CXCL8, IL6, TNF, VEGFA
IL10 signaling	1.23 × 10^−4^	3	IL10, IL6, TNF
IL8 signaling	1.73 × 10^−4^	4	CXCL1, CXCL8, ICAM1, VEGFA
**Cellular Immune Response**			
Role of cytokines in mediating communication between immune cells	8.74 × 10^−11^	6	CSF2, CXCL8, IL10, IL6, TGFB1, TNF
Th1 and Th2 activation pathway	8.01 × 10^−5^	4	ICAM1, IL10, IL6, TGFB1
Th1 pathway	5.81 × 10^−4^	3	ICAM1, IL10, IL6
Th2 pathway	8.13 × 10^−4^	3	ICAM1, IL10, TGFB1
Communication between innate and adaptive immune cells	7.44 × 10^−3^	5	CSF2, CXCL8, IL10, IL6, TNF
**Diseases or Functions Annotations**			
**Antimicrobial Response, Inflammatory Response**			
Antibacterial response	1.66 × 10^−22^	13	CAMP, CCL20, DEFB1, DEFB103A/DEFB103B, DEFB4A/DEFB4B, IL10, IL6, RNASE7, S100A12, S100A7, S100A8, S100A9, TNF
**Antimicrobial Response, Inflammatory Response**			
Chemoattraction	5.09 × 10^−23^	12	CAMP, CCL2, CCL20, CSF2, CXCL1, CXCL3, CXCL8, DEFB4A/DEFB4B, FN1, TGFB1, TNF, VEGFA
**Cellular Movement, Hematological System Development and Function, Immune Cell Trafficking, Inflammatory Response**			
Chemotaxis	4.28 × 10^−37^	27	CAMP, CCL2, CCL20, CSF2, CXCL1, CXCL2, CXCL3, CXCL8, DEFB1, DEFB103A/DEFB103B, DEFB4A/DEFB4B, FGF2, FN1, ICAM1, IL10, IL6, S100A12, S100A7, S100A8, S100A9, SELE, SMAD3, SMAD4, TGFA, TGFB1, TNF, VEGFA
Chemotaxis of leukocytes	1.43 × 10^−35^	24	CAMP, CCL2, CCL20, CSF2, CXCL1, CXCL2, CXCL3, CXCL8, DEFB1, DEFB103A/DEFB103B, DEFB4A/DEFB4B, FN1, ICAM1, IL10, IL6, S100A12, S100A7, S100A8, S100A9, SELE, SMAD3, TGFB1, TNF, VEGFA
**Inflammatory Response**			
Inflammatory response	9.34 × 10^−29^	26	CAMP, CCL2, CCL20, CSF2, CXCL1, CXCL2, CXCL3, CXCL8, DEFB1, DEFB103A/DEFB103B, DEFB4A/DEFB4B, FGF1, FGF2, FN1, ICAM1, IL10, IL6, S100A12, S100A7, S100A8, S100A9, SELE, SMAD3, TGFB1, TNF, VEGFA
Proinflammatory response	4.92 × 10^−15^	7	CCL2, CXCL3, CXCL8, IL10, IL6, TNF, VEGFA
Innate immune response	5.94 × 10^−14^	10	CAMP, CXCL1, CXCL8, FN1, IL10, IL6, RNASE7, S100A12, SMAD3, TNF
**Tissue Development**			
Healing of wound	1.53 × 10^−20^	13	CSF2, FGF1, FGF2, FN1, ICAM1, IL10, IL6, SMAD3, SMAD4, TGFA, TGFB1, TNF, VEGFA
**Cell-To-Cell Signaling and Interaction, Cellular Movement, Hematological System Development and Function, Immune Cell Trafficking, Inflammatory Response**			
Cell movement of monocytes	1.37 × 10^−31^	19	CAMP, CCL2, CCL20, CSF2, CXCL3, CXCL8, DEFB1, DEFB103A/DEFB103B, FN1, ICAM1, IL10, IL6, S100A12, S100A7, SELE, SMAD3, TGFB1, TNF, VEGFA
Cell movement of neutrophils	2.01 × 10^−28^	21	CAMP, CCL2, CSF2, CXCL1, CXCL2, CXCL3, CXCL8, DEFB1, DEFB103A/DEFB103B, DEFB4A/DEFB4B, FN1, ICAM1, IL10, IL6, S100A12, S100A8, S100A9, SELE, SMAD3, TGFB1, TNF
**Cell-To-Cell Signaling and Interaction, Cellular Movement, Hematological System Development and Function, Immune Cell Trafficking, Inflammatory Response**			
Recruitment of cells	7.85 × 10^−29^	21	BMP10, CAMP, CCL2, CCL20, CSF2, CXCL1, CXCL2, CXCL3, CXCL8, DEFB4A/DEFB4B, FGF2, FN1, ICAM1, IL10, IL6, S100A8, SELE, SMAD3, TGFB1, TNF, VEGFA
Recruitment of leukocytes	1.05 × 10^−25^	19	BMP10, CAMP, CCL2, CCL20, CSF2, CXCL1, CXCL2, CXCL3, CXCL8, DEFB4A/DEFB4B, FN1, ICAM1, IL10, IL6, S100A8, SELE, SMAD3, TGFB1, TNF
**Cell-To-Cell Signaling and Interaction, Inflammatory Response**			
Immune response of cells	2.70 × 10^−18^	18	CAMP, CCL2, CCL20, CSF2, CXCL1, CXCL3, CXCL8, FN1, ICAM1, IL10, IL6, S100A12, S100A8, S100A9, SMAD3, TGFB1, TNF, VEGFA
Immune response of myeloid cells	2.85 × 10^−17^	13	CAMP, CCL2, CSF2, CXCL1, CXCL3, CXCL8, FN1, ICAM1, IL10, IL6, S100A9, TGFB1, TNF
**Cellular Growth and Proliferation**			
Angiogenesis	1.74 × 10^−23^	24	BMP10, CAMP, CCL2, CSF2, CXCL1, CXCL2, CXCL8, FGF1, FGF2, FN1, ICAM1, IL10, IL6, PIGF, S100A12, S100A8, S100A9, SELE, SMAD3, SMAD4, TGFA, TGFB1, TNF, VEGFA
Proliferation of vascular cells	3.32 × 10^−20^	16	CAMP, CCL2, CXCL1, CXCL8, FGF1, FGF2, FN1, IL10, IL6, S100A8, S100A9, SMAD3, SMAD4, TGFB1, TNF, VEGFA
**Cellular Movement, Hematological System Development and Function, Immune Cell Trafficking**			
Cell survival	1.73 × 10^−17^	24	CAMP, CCL2, CSF2, CXCL1, CXCL2, CXCL3, CXCL8, DEFB103A/DEFB103B, DEFB4A/DEFB4B, FGF1, FGF2, FN1, ICAM1, IL10, IL6, S100A8, S100A9, SELE, SMAD3, SMAD4, TGFA, TGFB1, TNF, VEGFA
Cell viability	2.74 × 10^−15^	22	CAMP, CCL2, CSF2, CXCL1, CXCL2, CXCL3, CXCL8, FGF1, FGF2, FN1, ICAM1, IL10, IL6, S100A8, S100A9, SELE, SMAD3, SMAD4, TGFA, TGFB1, TNF, VEGFA

## Data Availability

Morio et al. reported the concentrations of 13 CCBMs in tissue culture media of HEPM cells and gingival fibroblasts at 0, 24, and 48 h after treatment with 255, 405, or 255/405 UV irradiation [6]. In the current study, we used these CCBM values as a subset of data to assess the ability of UV irradiation to activate or inhibit cellular pathways related to immune functions.

## Supplementary Materials

The following supporting information can be downloaded at: https://www.mdpi.com/article/10.3390/pharmaceutics14091979/s1, Supplementary Figure S1: A schematic diagram of UV-induced MAPK signaling; Supplementary Figure S2: A schematic diagram of neuropathic pain signaling; Supplementary Table S1: Antimicrobial peptides (AMPs) expressed or secreted in cells, tissues, and tissue explants after irradiation with UVC, UVB, or UVA; Supplementary Table S2: Chemokines, cytokines, and biomarkers (CCBMs) expressed or secreted in cells, tissues, and tissue explants after irradiation with UVC, UVB, or UVA; Supplementary Table S3: Antimicrobial peptides and biomarkers in the literature dataset (*n* = 32) participating in the activation of innate and immune mechanisms, as determined using IPA canonical pathways annotations; and Supplementary Table S4: Antimicrobial peptides and biomarkers in the literature dataset (*n* = 32) participating in the activation of innate and immune mechanisms as determined using IPA diseases and function annotations. Refs. [52,53,54,55,56,57,58,59,60,61,62,63,64,65,66,67,68,69,70,71] are cited in Supplementary Materials.
