# Supplementary material for: Antimicrobial Peptides and Biomarkers Induced by Ultraviolet Irradiation Have the Potential to Reduce Endodontic Inflammation and Facilitate Tissue Healing
Source: Pharmaceutics. 2022 Sep 19;14(9):1979. doi: 10.3390/pharmaceutics14091979 (PMC9503046; doi:10.3390/pharmaceutics14091979)
Supplement: Supplementary file 1 [file pharmaceutics-14-01979-s001.zip › pharmaceutics-1897757-supplementary/Supplementary Figure S1.pdf]

Supplementary Figure S1. A schematic diagram of UV-induced MAPK signaling. This schematic diagram was prepared using Ingenuity Pathway Analysis software (IPA, Qiagen, Germantown, MD) and shows the overlays and effects that UVC, UVB, and UVA irradiation can have on cellular DNA, cellular receptors, signal transduction pathways, and cellular responses [49,52–54].

DNA damage. UVC and UVA can damage DNA directly if conditions and dose permit. UVC can damage DNA, which affects ATR and signals to TP53. UVA can cause reactive oxygen species (ROS) and damage DNA affecting ATM, which signals to JNK and then to TP53. TP53 influences cell cycle arrest and cell apoptosis.

MAPK signaling. MAPK signaling occurs through the p38 MAPK, the extracellular signal-regulated kinase 1/2 (ERK), and the c-Jun N-terminal kinase (JNK) pathways. All three are targets for UVC, UVB, and UVA, which affects the regulation and outcome of cell responses including cellular apoptosis, cellular proliferation, and tumor suppression.

P38 MAPK signaling. The p38 MAPK pathway regulates a variety of cellular processes that includes cell apoptosis and the release of cytokines by macrophages and neutrophils. P38 MAPK is stimulated via a number of paths. UVA and UVB stimulate phosphorylation of p38 MAPK. UVA, UVB, and UVC stimulates EGFR signaling to p38 MAPK. Activated p38 MAPK in turn signals on to different paths. One path is through MTOR, which regulates cell proliferation, autophagy, and apoptosis and UVB induced changes in MTOR impacts the phosphorylation of EIF4EBP1 causing dissociation to EIF4E. In a second path, p38 MAPK increases STAT1. In a third path, p38 MAPK signals to TP53. Finally, p38 MAPK signals through RPS6KA5 to BCL2 associated agonist of cell death (BAD), which is involved in cell survival.

ERK signaling. The ERK1/2 pathway regulates cellular processes that include cell proliferation, cell differentiation, and cell survival; apoptosis; and stress responses. ERK is stimulated via a number of paths. UVC activates SRC signaling through RAS, raf, and MAP2K1 to ERK. UVB activates PKC and PI3K via PKC to ERK. UVA activates EGFR and signals through PI3K to ERK. UVA signals to PLC to Ca^{2+} to PRKCA through RAS, Raf, and MAP2K1 to ERK. Activated ERK in turn signals on to different paths. In one path, UVC and UVB signals from ERK to transcription factors c-Jun and c-Fos (AP-1) resulting in the expression of molecules that influence cellular proliferation and tumor suppression. In a second path, UVB signals go on to RPS6KA3 to BAD, which is involved in cell survival. In a third path, ERK signals through RPS6KA5 to MTOR and Histone H3, which is related to chromatin remodeling. Finally, ERK signals to Rsk.

JNK signaling. The JNK pathway regulates cellular processes that include cell proliferation and apoptosis. Signaling through JNK to transcription factors c-Jun and c-Fos (AP-1) results in the expression of molecules that influence cellular proliferation and tumor suppression. JNK is stimulated via a number of paths. UVA activates JNK directly. UVC signals to PKC and on to JNK and UVC and UVA activate sphingomyelinase, which activates JNK. UVB signals from PKC through JNK and UVA signals to PLC to Ca^{2+} to PRKCA to RAS to JNK. Activated (phosphorylated) JNK in turn signals on to different paths. One path is on to transcription factors c-Jun and c-Fos (AP-1) resulting in the expression of molecules that influence cellular proliferation and tumor suppression. A second path is to TP53 and UVB-induced signaling pathways also lead to stabilization and phosphorylation of p53. Finally, JNK signals to Rsk and STAT1, a transcription regulator.

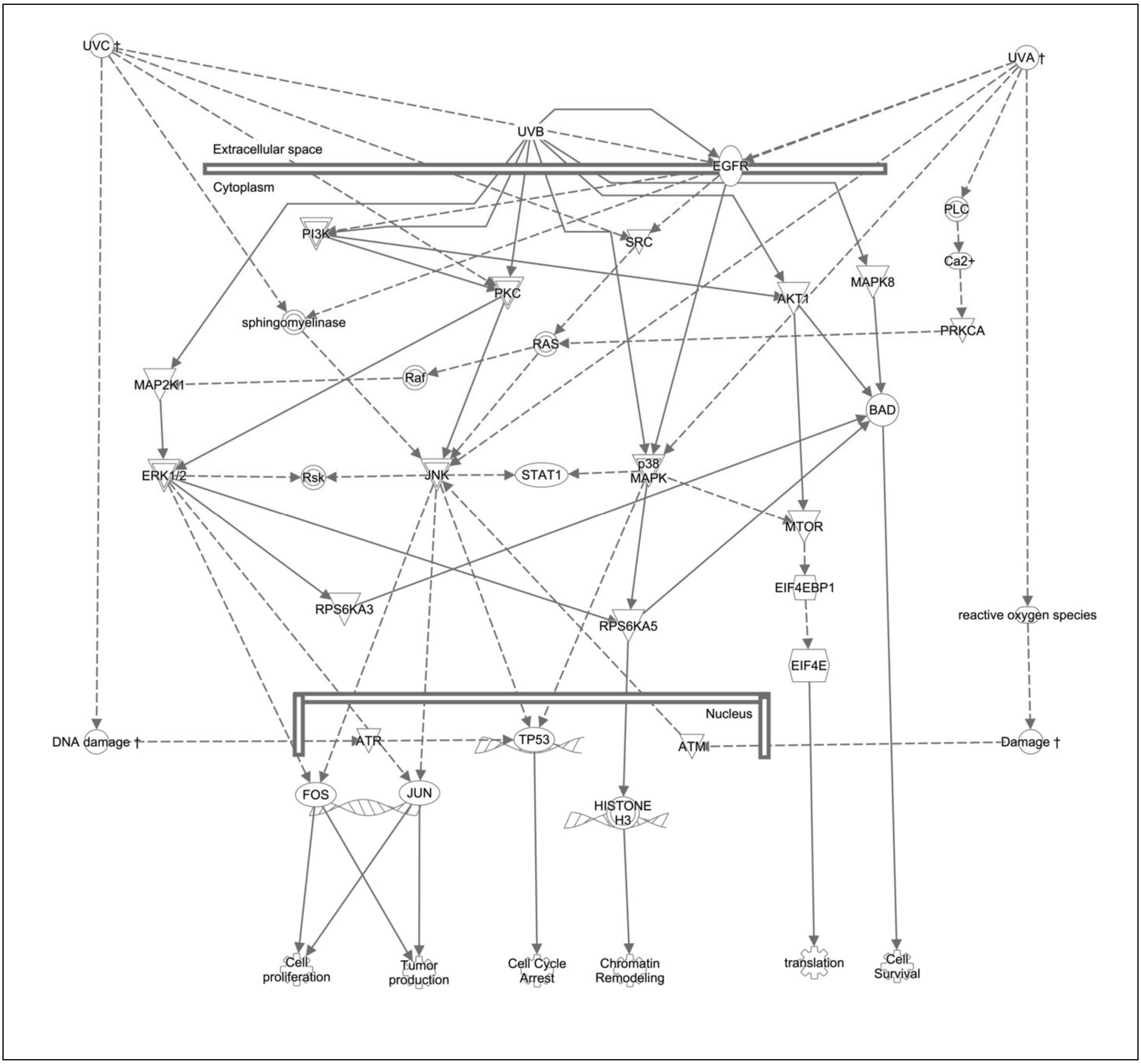
UVC †
UVA †
UVB
Extracellular space
Cytoplasm
EGFR
PI3K
SRC
PLC
Ca2+
PKC
AKT1
MAPK8
sphingomyelinase
PRKCA
RAS
Raf
MAP2K1
BAD
ERK1/2
Rsk
JNK
STAT1
p38 MAPK
MTOR
EIF4EBP1
reactive oxygen species
RPS6KA3
RPS6KA5
EIF4E
Nucleus
DNA damage †
ATR
TP53
ATM
Damage †
FOS
JUN
HISTONE H3
Cell proliferation
Tumor production
Cell Cycle Arrest
Chromatin Remodeling
translation
Cell Survival
