# Supplementary material for: Antimicrobial Peptides and Biomarkers Induced by Ultraviolet Irradiation Have the Potential to Reduce Endodontic Inflammation and Facilitate Tissue Healing
Source: Pharmaceutics. 2022 Sep 19;14(9):1979. doi: 10.3390/pharmaceutics14091979 (PMC9503046; doi:10.3390/pharmaceutics14091979)

Supplementary Figure S2. A schematic diagram of neuropathic pain signaling. This schematic diagram was prepared using Ingenuity Pathway Analysis software (IPA, Qiagen, Germantown, MD). Many oral related infections, inflammation, and tissue injury/peripheral nerve injury can be stimuli that activate receptors on the surface of cells initiating signal transduction through MAPK signaling pathways. UV irradiation may alter signaling through ERK and p38 MAPK pathways and thus be potential therapeutic strategies for pain management [50,51].

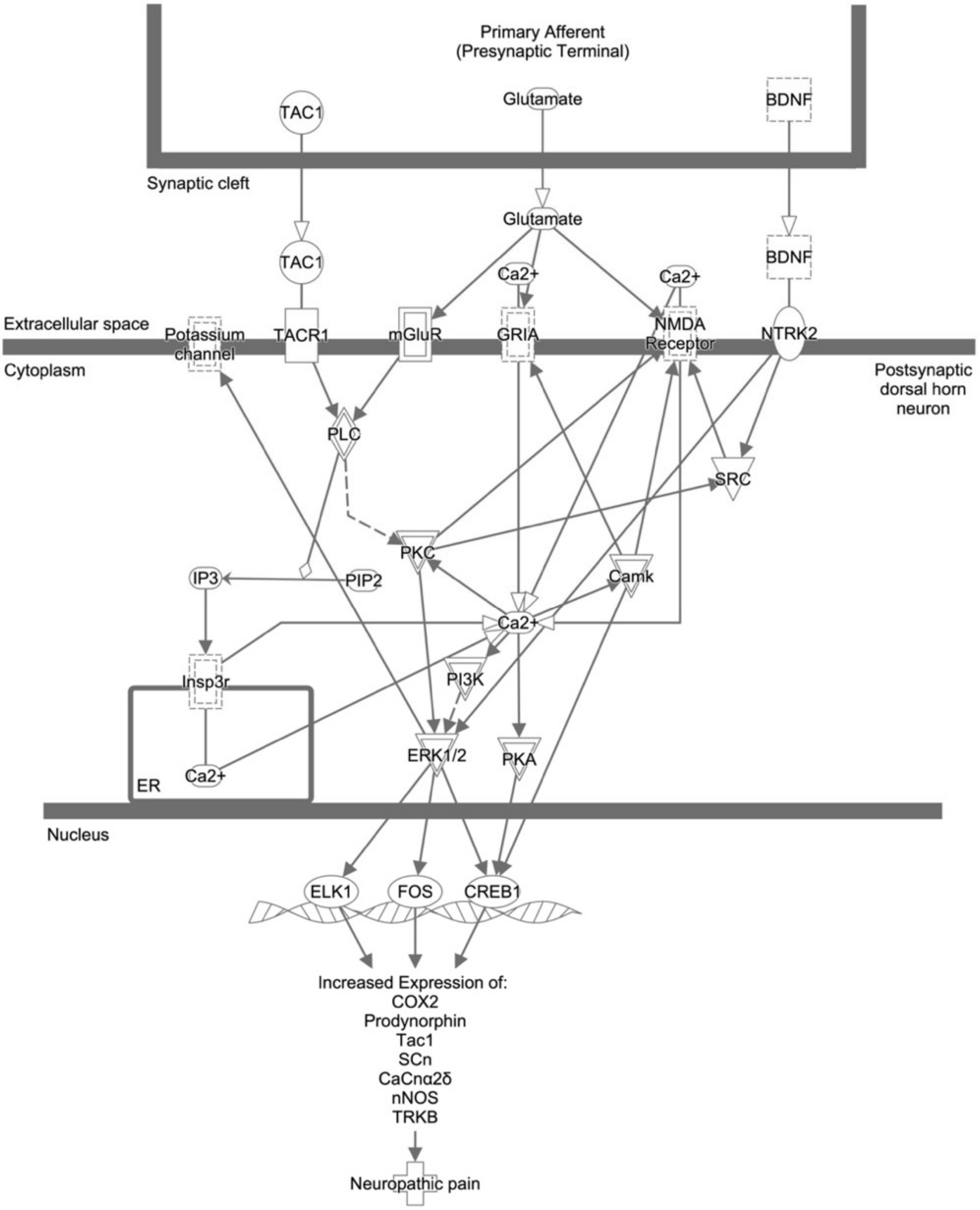

Supplement: Supplementary file 1 [file pharmaceutics-14-01979-s001.zip › pharmaceutics-1897757-supplementary/Supplementary Figure S2.pdf]
