# Supplementary material for: Antimicrobial Peptides and Biomarkers Induced by Ultraviolet Irradiation Have the Potential to Reduce Endodontic Inflammation and Facilitate Tissue Healing
Source: Pharmaceutics. 2022 Sep 19;14(9):1979. doi: 10.3390/pharmaceutics14091979 (PMC9503046; doi:10.3390/pharmaceutics14091979)
Supplement: Supplementary file 1 [file pharmaceutics-14-01979-s001.zip › pharmaceutics-1897757-supplementary/Supplementary Table S1.pdf]

Supplementary Table S1. Antimicrobial peptides (AMPs) expressed or secreted in cells, tissues, and tissue explants after irradiation with UVC, UVB, or UVA.

Wavelength (nm)	AMP	Dose(s)	Cells/tissues	Outcome (reference)
**UVC spectrum (200 to 290 nm)**				
280-315	CAMP	12-40 mJ/cm^2	Skin	• Biopsies from human skin had increased levels of hCAP18/LL37 mRNA expression at 24-28 hours after exposure [20]
**UVB Spectrum (290 to 320 nm)**				
311[a]	CAMP	1.6 J/cm^2 [b]	Skin	• Biopsies from human atopic skin had no changes in levels of LL37 mRNA expression after exposure [23]
311[a]	DEFB1	1.6 J/cm^2 [b]	Skin	• Biopsies from human atopic skin had increased levels of HBD1 mRNA expression after exposure [23]
311[a]	DEFB103A	1.6 J/cm^2 [b]	Skin	• Biopsies from human atopic skin had no changes in levels of HBD3 mRNA expression after exposure [23]
311[a]	DEFB4A	1.6 J/cm^2 [b]	Skin	• Biopsies from human atopic skin had decreased levels of HBD2 mRNA expression after exposure [23]
312	DEFB4A	2 MED	Skin	• Biopsies from human skin had increased levels of HBD2 expression (IHC) at 24 hours after exposure [28]
312	PI3	2 MED	Skin	• Biopsies from human skin had increased levels of elafin expression (IHC) at 24 hours after exposure [28]
312	S100A12	2 MED	Skin	• Biopsies from human skin had increased levels of S100A12 expression (IHC) at 24 hours after exposure [28]
312	S100A7	2 MED	Skin	• Biopsies from human skin had increased levels of S100A7 expression (IHC) at 24 hours after exposure [28]
312	S100A8/9	2 MED	Skin	• Biopsies from human skin had increased levels of S100A8/9 expression (IHC) at 24 hours after exposure [28]
313[c]	DEFB103A	0, 150, 350 J/m^2	Keratinocytes	• Human keratinocytes had increased levels of HBD3 mRNA expression and secretion at 350 J/m^2 in 24 and 48 hours after exposure [21]

313[c]	DEFB103A	3 X 1 MED[d]	Skin	• Biopsies from human skin had increased levels of HBD3 expression (IHC) in 3/3 individuals and present in skin washing fluids (ELISA) at days 1 and 6 [21]
313[c]	DEFB103A	250 J/m^2	Skin explants	• Human skin explants had increased HBD3 expression (IHC) at 24 hours after exposure [21]
313[c]	DEFB4A	0, 150, 350 J/m^2	Keratinocytes	• Human keratinocytes had increased levels of HBD2 mRNA expression and secretion at 350 J/m^2 in 24 and 48 hours after exposure [21]
313[c]	DEFB4A	3 X 1 MED[d]	Skin	• Biopsies from human skin had increased levels of HBD2 expression (IHC) in 1/3 individuals, but low concentrations in skin washing fluids (ELISA) at days 1 and 6 [21]
313[c]	DEFB4A	250 J/m^2	Skin explants	• Human skin explants had minimal HBD2 expression (IHC) at 24 hours after exposure [21]
313[c]	RNASE7	0, 150, 350 J/m^2	Keratinocytes	• Human keratinocytes had increased levels of RNase7 mRNA expression and secretion at 350 J/m^2 in 24 and 48 hours after exposure [21]
313[c]	RNASE7	3 X 1 MED[d]	Skin	• Biopsies from human skin had increased levels of RNAse7 expression (IHC) in 3/3 individuals and present in skin washing fluids (ELISA) at days 1 and 6 [21]
313[c]	RNASE7	250 J/m^2	Skin explants	• Human skin explants had increased RNAse7 expression in skin explants (IHC) at 24 hours after exposure [21]
313[c]	S100A7	0, 150, 350 J/m^2	Keratinocytes	• Human keratinocytes had increased levels of psoriasin mRNA expression and secretion at 350 J/m^2 in 24 and 48 hours after exposure [21]
313[c]	S100A7	3 X 1 MED[d]	Skin	• Biopsies from human skin had increased levels of psoriasin (IHC) in 3/3 individuals and present in skin washing fluids (ELISA) at days 1 and 6 [21]
313[c]	S100A7	250 J/m^2	Skin explants	• Human skin explants had increased levels of psoriasin expression (IHC) at 24 hours after exposure [21]
313[e]	DEFB1	30, 100 mJ/cm^2	Keratinocytes	• Neonatal foreskin, HaCaT, and A431 keratinocytes all had increased levels of HBD1 mRNA expression at 12 hours after exposure [55]
313[e]	DEFB4A	30, 100 mJ/cm^2	Keratinocytes	• Neonatal foreskin, HaCaT, and A431 keratinocytes all had increased levels of HBD2 mRNA expression at 12 hours after exposure [55]
**UVA spectrum (320 to 400 nm)**				

340-400	CAMP	20 J/m^2 [f]	Skin	• Biopsies from human skin did not have changes in hCAP18 mRNA expression after exposure [23]
340-400	DEFB1	20 J/m^2 [f]	Skin	• Biopsies from human sclerotic skin had decreased levels of HBD1 mRNA expression at 20 J/m^2 (800 J/m^2 total), but not in unaffected skin [22]
340-400	DEFB103A	20 J/m^2 [f]	Skin	• Biopsies from human sclerotic skin had decreased levels of HBD3 mRNA expression at 20 J/m^2 (800 J/m^2 total), but not in unaffected skin [22]

[a]UV range from 310 to 315 nm, emission peak at 311 nm
[b]Dose of 0.1 to 0.2 J/cm^2 per each of 6 sessions, 1.6 J/cm^2 total
[c]UV range from 290 to 320 nm, emission peak at 313 nm
[d]UV irradiated on 3 consecutive days with 1 MED
[e]UV range from 280 to 320 nm, emission peak at 313 nm
[f]Dose of 20 J/cm^2 per each of 40 sessions, 800 J/cm^2 total

ELISA, enzyme-linked immunosorbent assay
FACS, fluorescence-activated cell sorting
IF, immunofluorescence
IHC, Immunohistochemistry
MED, minimal erythema dose
