# Supplementary material for: Antimicrobial Peptides and Biomarkers Induced by Ultraviolet Irradiation Have the Potential to Reduce Endodontic Inflammation and Facilitate Tissue Healing
Source: Pharmaceutics. 2022 Sep 19;14(9):1979. doi: 10.3390/pharmaceutics14091979 (PMC9503046; doi:10.3390/pharmaceutics14091979)
Supplement: Supplementary file 1 [file pharmaceutics-14-01979-s001.zip › pharmaceutics-1897757-supplementary/Supplementary Table S2.pdf]

Supplementary Table S2. Chemokines, cytokines, and biomarkers (CCBMs) expressed or secreted in cells, tissues, and tissue explants after irradiation with UVC, UVB, or UVA.

Wavelength (nm)	CCBM	Dose(s)	Cells/tissues	Outcome (reference)
**UVC spectrum (200 to 290 nm)**				
255 / 405	FGF1	9.28 mJ/cm²/ 17.88 mJ/cm²	Fibroblasts	• Fibroblasts had increased levels of FGF-acidic secretion at 24 hours after exposure [6]
255 / 405	FGF1	9.28 mJ/cm²/ 17.88 mJ/cm²	HEPM cells	• HEPM cells had decreased levels of FGF-acidic secretion at 48 hours after exposure [6]
255 / 405	IL6	9.28 mJ/cm²/ 17.88 mJ/cm²	Fibroblasts	• Fibroblasts had increased levels of IL6 secretion at 24 and 48 hours after exposure [6]
255 / 405	IL6	9.28 mJ/cm²/ 17.88 mJ/cm²	HEPM cells	• HEPM cells had increased levels of IL6 secretion at 24 hours after exposure [6]
255 / 405	PIGF	9.28 mJ/cm²/ 17.88 mJ/cm²	Fibroblasts	• Fibroblasts had increased levels of PIGF secretion at 24 hours after exposure [6]
255 / 405	PIGF	9.28 mJ/cm²/ 17.88 mJ/cm²	HEPM cells	• HEPM cells had decreased levels of PIGF secretion at 24 hours after exposure [6]
255 / 405	TGFA	9.28 mJ/cm²/ 17.88 mJ/cm²	Fibroblasts	• Fibroblasts had increased levels of TGFA secretion at 48 hours after exposure [6]
255 / 405	TNFα	9.28 mJ/cm²/ 17.88 mJ/cm²	HEPM cells	• HEPM cells had increased levels of TNFA secretion at 48 hours after exposure [6]
255 / 405	VEGF	9.28 mJ/cm²/ 17.88 mJ/cm²	Fibroblasts	• Fibroblasts had decreased levels of VEGF secretion at 24 hours after exposure [6]
255 / 405	VEGF	9.28 mJ/cm²/ 17.88 mJ/cm²	HEPM cells	• HEPM cells had decreased levels of VEGF secretion at 24 hours after exposure [6]
254	FGF	15-60 mJ/cm²	Skin	• Epidermal wounds in rats had increased levels of FGF expression [56]
254	FN1		Fibroblasts	• Human fibroblasts had increased levels of fibronectin secretion and collagen lattices contain treated fibroblasts contracted faster at 7 days [12]
254	TGFB	15-60 mJ/cm²	Skin	• Epidermal wounds in rats had increased levels of TGFB mRNA expression and secretion at 7 (15 mJ/cm²) and 21 (60 mJ/cm²) days after exposure [57]
255	BMP10	9.28 mJ/cm²	HEPM cells	• HEPM cells had decreased levels of BMP-10 secretion at 48 hours after exposure [6]

255	FGF1	9.28 mJ/cm²	HEPM cells	• HEPM cells had decreased levels of FGF-acidic secretion at 24 hours after exposure [6]
255	IL6	9.28 mJ/cm²	Fibroblasts	• Fibroblasts had increased levels of IL6 secretion at 24 and 48 hours after exposure [6]
255	IL6	9.28 mJ/cm²	HEPM cells	• HEPM cells had increased levels of IL6 secretion at 24 and 48 hours after exposure [6]
255	PIGF	9.28 mJ/cm²	Fibroblasts	• Fibroblasts had increased levels of PIGF secretion at 24 hours after exposure [6]
255	TGFA	9.28 mJ/cm²	Fibroblasts	• Fibroblasts had increased levels of TGFA secretion at 24 hours after exposure [6]
255	VEGF	9.28 mJ/cm²	Fibroblasts	• Fibroblasts had decreased levels of VEGF secretion at 24 hours after exposure [6]
255	VEGF	9.28 mJ/cm²	HEPM cells	• HEPM cells had increased levels of VEGF secretion at 24 hours after exposure [6]
200-320 a	CSF	300 J/m²	Keratinocytes	• Murine PAM212 cells had increased levels of GM-CSF mRNA expression and secretion as early as 1 hour after exposure [58]
200-320 a	IL3	300 J/m²	Keratinocytes	• Murine PAM212 cells had increased levels of IL3 mRNA expression as early as at 1 hour after exposure [58]
**UVB Spectrum (290 to 320 nm)**				
250-400 i	IL6	100 J/m²	Keratinocytes	• Human epidermal cells had increased levels of IL6 mRNA expression and secretion at 24 hours after exposure [59]
250–360 h	IL3	120-400 J/m²	Keratinocytes	• Murine PAM 212 cells had increased levels of IL3 expression and secretion after exposure [60]
280-315	IL6	250-500 J/m²	Keratinocytes	• NHEK had increased levels of IL6 expression and secretion at 24 hours after exposure [61]
280-315	TNFA	250-500 J/m²	Keratinocytes	• NHEK had increased levels of TNFα expression and secretion at 24 hours after exposure [61]
280-320	VEGF	30-60 mJ/cm²	Skin	• Biopsies from human and murine skin had increased levels of VEGF immunostaining at 48 hours after exposure [26]
301-312 k	ICAM1	100 mJ/cm²	Endothelial cells	• Human dermal microvascular endothelial cell (HDMEC) had increased levels of

				ICAM-1 expression at 24 hours after exposure [62]
305 e	IL1	0.1 J/cm²	Keratinocytes	• Human keratinocytes had increased levels of IL1 secretion at 4 days after exposure [25]
310	IL1	100-800 mJ/cm²	Keratinocytes	• Human squamous cell carcinoma, murine Pam 212, and murine epidermal cell keratinocytes had increased levels of IL1 mRNA expression and secretion at 24 hours after exposure [63–65]
310 g	IL1	184 mJ/cm²	Skin	• Biopsies from human skin had increased levels of IL1 immunostaining at 24 hours after exposure [27]
310 g	TNFA	184 mJ/cm²	Skin	• Biopsies from human skin had increased levels of TNF immunostaining at 24 hours after exposure [27]
310 j	SELE	2.5 MED	Skin	• Biopsies from human skin had increased levels of E-selectin immunostaining at 4, 8, 24 hours after exposure [27]
310 j	ICAM1	15-20 mJ/cm²	Endothelial cells	• Human dermal microvascular endothelial cell (HMEC-1) line had increased levels of ICAM-1 staining expression at 24 hours after exposure [66]
310 j	ICAM1	2.5 MED	Skin	• Biopsies from human skin had increased levels of ICAM-1 immunostaining at 4, 8, 24 hours after exposure [27]
312	CCL2	2 MED	Skin	• Biopsies from human skin had increased levels of CCL2 mRNA expression at 24 hours after exposure [28]
312	CCL20	2 MED	Skin	• Biopsies from human skin had increased levels of CCL20 mRNA expression at 24 hours after exposure [28,67]
312	CXCL1	2 MED	Skin	• Biopsies from human skin had increased levels of CXCL1 mRNA expression at 24 hours after exposure [28]
312	CXCL8	2 MED	Skin	• Biopsies from human skin had increased levels of IL8 mRNA expression at 24 hours after exposure [28]
312	IL10	2 MED	Skin	• Biopsies from human skin had increased levels of IL10 mRNA expression at 24 hours after exposure [28]
313 b	IL6	10-100 J/cm²	Keratinocytes	• Normal and malignant human keratinocytes had increased levels of IL6 mRNA

				expression and secretion at 24 hours after exposure [68]
313 f	TNFA	200-750 J/m²	Keratinocytes	• Human HaCaT cells had increased levels of TNFA secretion at 24 hours after exposure [69]
313 f	TNFA	200-750 J/m²	Skin	• Murine epidermis and dermis had increased levels of TNFA expression at 12 hours after exposure [69]
NB-UVB	SMAD3	1.5-1.8 J/cm²	Skin	• Biopsies from skin of individuals with atopic dermatitis had increased levels of Smad3 mRNA expression at 12 hours after exposure [70]
NB-UVB	SMAD4	1.5-1.8 J/cm²	Skin	• Biopsies from skin of individuals with atopic dermatitis had increased levels of Smad4 mRNA expression at 12 hours after exposure [70]
NB-UVB	TGFB	1.5-1.8 J/cm²	Skin	• Biopsies from skin of individuals with atopic dermatitis had increased levels of TGFB mRNA expression at 12 hours after exposure [70]
UVB	TGFA	3 MED	Skin	• Cutaneous exudates from human skin abrasions had increased levels of TGFA in irradiated sites at 30 minutes after exposure [71]
**UVA spectrum (320 to 400 nm)**				
340-400	CXCL8	20 J/m²	Skin	• Sclerotic skin biopsies had increased levels of IL8 mRNA expression (20 J/m², cumulative 800 J/m² total) that decreased after irradiation [22]
340-400	IL6	20 J/m²	Skin	• Sclerotic skin biopsies had increased levels of IL6 mRNA expression (20 J/m², cumulative 800 J/m² total) that decreased after irradiation [22]
360 c	CSF	1-10 J/cm²	Keratinocytes	• Human HaCaT keratinocytes had increased levels of GM-CSF mRNA expression and secretion at 24 and 48 hours after exposure through an IL1 alpha independent pathway [24]
365 d	CSF	1 J/cm²	Keratinocytes	• Human keratinocytes had increased levels of GM-CSF secretion at 4 days after exposure [25]

365 d	IL6	1 J/cm2	Keratinocytes	• Human keratinocytes had increased levels of IL6 secretion at 4 days after exposure [25]
365 d	IL8	1 J/cm2	Keratinocytes	• Human keratinocytes had increased levels of IL8 secretion at 4 days after exposure [25]
405	FGF1	17.88 mJ/cm2	Fibroblasts	• Fibroblasts had decreased levels of FGF-acidic secretion at 24 hours after exposure [6]

[a]UV range from 290 to 320 nm

[b]UV range from 290 to 320 nm, emission peak at 313 nm

[c]UV range from 330 to 460 nm, emission peak at 360 nm

[d]UV range from 310 to 400 nm, emission peak at 365 nm

[e]UV range from 295 to 315 nm, emission peak at 305 nm

[f]UV range from 290 to 320 nm, emission peak at 313 nm

[g]UV range from 270 to 380 nm, emission peak at 310 nm

[h]UV range from 250 to 360 nm; 65% in range of 280 to 315 nm (UVB); remaining in range of 250 to 280 nm (UVC) and in range of 315 to 360 nm (UVA)

[i]UV range from 250 to 400 nm

[j]UV range from 270 to 400 nm, emission peak at 310 nm; 43.3% in range of 290 to 320 nm (UVB); <0.1% in range of 200-290 nm (UVC); and 56.6% in range of 320-400 nm (UVA)

[k]UV range of 290 to 325 nm, emission peaks at 301 and 312 nm

MED, minimum erythemal dose
