# Supplementary material for: Antimicrobial Peptides and Biomarkers Induced by Ultraviolet Irradiation Have the Potential to Reduce Endodontic Inflammation and Facilitate Tissue Healing
Source: Pharmaceutics. 2022 Sep 19;14(9):1979. doi: 10.3390/pharmaceutics14091979 (PMC9503046; doi:10.3390/pharmaceutics14091979)
Supplement: Supplementary file 1 [file pharmaceutics-14-01979-s001.zip › pharmaceutics-1897757-supplementary/Supplementary Table S3.pdf]

Supplementary Table S3. Antimicrobial peptides and biomarkers in the literature dataset (n=32) participating in the activation of innate and immune mechanisms as determined using IPA Canonical Pathways annotations. Ingenuity Pathway Analysis (IPA, Qiagen, Germantown, MD) was used to assess whether the antimicrobial peptides and biomarkers in the literature dataset (n=32) would participate in the activation of innate and immune mechanisms. IPA Core Analysis was run on the list of 32 biomarkers and IPA Canonical Pathways annotations were associated with cellular stress and injury (e.g., wound healing); cytokine signaling; growth factor signaling; intracellular and second messenger signaling; cellular growth, proliferation, and development; cellular immune response; and organismal growth and development.

Canonical Pathways	-log (p-value)	p-value	No. of molecules	Identification of molecules
**Cellular stress and injury**				
Wound Healing Signaling Pathway	10.492	3.22E-11	9	CSF2, CXCL8, FGF2, FN1, IL6, TGFA, TGFB1, TNF, VEGFA
CLEAR Signaling Pathway	4.440	3.63E-05	5	FGF2, TGFA, TGFB1, TNF, VEGFA
**Cytokine signaling**				
IL17 Signaling Pathway	19.074	8.43E-20	13	CCL2, CCL20, CSF2, CXCL1, CXCL3, CXCL8, DEFB1, DEFB103A/DEFB103B, DEFB4A/DEFB4B, IL6, TGFB1, TNF, VEGFA
IL13 Signaling Pathway	6.345	4.52E-07	5	DEFB1, DEFB103A/DEFB103B, DEFB4A/DEFB4B, IL10, TGFB1
IL6 Signaling Pathway	4.597	2.53E-05	4	CXCL8, IL6, TNF, VEGFA
IL12 Signaling Pathway and Production in Macrophages	4.493	3.21E-05	4	IL10, S100A8, TGFB1, TNF
Th1 and Th2 Activation Pathway	4.096	8.01E-05	4	ICAM1, IL10, IL6, TGFB1
IL10 Signaling Pathway	3.910	1.23E-04	3	IL10, IL6, TNF
IL8 Signaling Pathway	3.762	1.73E-04	4	CXCL1, CXCL8, ICAM1, VEGFA
Th1 Pathway	3.236	5.81E-04	3	ICAM1, IL10, IL6
Th2 Pathway	3.090	8.13E-04	3	ICAM1, IL10, TGFB1
IL23 Signaling Pathway	2.759	1.74E-03	2	CSF2, TNF
Acute Phase Response Signaling	2.714	1.93E-03	3	FN1, IL6, TNF
NF-κB Signaling Pathway	0.752	2.85E-01	2	TGFA, TNF
**Growth factor signaling**				
TGF-β Signaling Pathway	3.541	2.88E-04	3	SMAD3, SMAD4, TGFB1
**Intracellular and second messenger signaling**				

NAD Signaling Pathway	4.316	4.83E-05	4	FGF2, TGFA, TGFB1, VEGFA
Protein Kinase A Signaling	1.764	1.72E-02	3	SMAD3, SMAD4, TGFB1
**Cellular growth, proliferation, and development**				
STAT3 Signaling Pathway	4.506	3.12E-05	4	FGF2, TGFA, TGFB1, VEGFA
ILK Signaling Pathway	2.618	2.41E-03	3	FN1, TNF, VEGFA
FGF Signaling Pathway	2.226	5.94E-03	2	FGF1, FGF2
**Cellular immune response**				
IL17 Signaling Pathway	19.074	8.43E-20	13	CCL2, CCL20, CSF2, CXCL1, CXCL3, CXCL8, DEFB1, DEFB103A/DEFB103B, DEFB4A/DEFB4B, IL6, TGFB1, TNF, VEGFA
TREM1 Signaling Pathway	15.208	6.19E-16	9	CCL2, CSF2, CXCL3, CXCL8, DEFB4A/DEFB4B, ICAM1, IL10, IL6, TNF
Agranulocyte Adhesion and Diapedesis	12.830	1.48E-13	10	CCL2, CCL20, CXCL1, CXCL2, CXCL3, CXCL8, FN1, ICAM1, SELE, TNF
Granulocyte Adhesion and Diapedesis	11.614	2.43E-12	9	CCL2, CCL20, CXCL1, CXCL2, CXCL3, CXCL8, ICAM1, SELE, TNF
HMGB1 Signaling Pathway	10.353	4.44E-11	8	CCL2, CSF2, CXCL8, ICAM1, IL6, SELE, TGFB1, TNF
Role of Cytokines in Mediating Communication between Immune Cells	10.058	8.74E-11	6	CSF2, CXCL8, IL10, IL6, TGFB1, TNF
Th17 Activation Pathway	5.532	2.94E-06	7	CCL20, CSF2, DEFB1, DEFB103A/DEFB103B, DEFB4A/DEFB4B, IL10, IL6
IL6 Signaling Pathway	4.597	2.53E-05	4	CXCL8, IL6, TNF, VEGFA
IL12 Signaling Pathway and Production in Macrophages	4.493	3.21E-05	4	IL10, S100A8, TGFB1, TNF
Th1 and Th2 Activation Pathway	4.096	8.01E-05	4	ICAM1, IL10, IL6, TGFB1
IL10 Signaling Pathway	3.910	1.23E-04	3	IL10, IL6, TNF
IL8 Signaling Pathway	3.762	1.73E-04	4	CXCL1, CXCL8, ICAM1, VEGFA
Th1 Pathway	3.236	5.81E-04	3	ICAM1, IL10, IL6
Th2 Pathway	3.090	8.13E-04	3	ICAM1, IL10, TGFB1
IL23 Signaling Pathway	2.759	1.74E-03	2	CSF2, TNF
PFKFB4 Signaling Pathway	2.724	1.89E-03	2	FGF2, TGFB1
Communication between Innate and Adaptive Immune Cells	2.128	7.44E-03	5	CSF2, CXCL8, IL10, IL6, TNF
p38 MAPK Signaling Pathway	1.947	1.13E-02	2	TGFB1, TNF

Antiproliferative Role of TOB in T Cell Signaling Pathway	1.719	1.91E-02	3	SMAD3, SMAD4, TGFB1
NF-κB Signaling Pathway	0.752	2.85E-01	2	TGFA, TNF
**Organismal growth and development**				
ID1 Signaling Pathway	11.393	4.05E-12	9	FGF2, FN1, IL6, S100A9, SMAD3, SMAD4, TGFB1, TNF, VEGFA
CSDE1 Signaling Pathway	4.237	5.79E-05	3	CCL2, FN1, TGFB1
BMP Signaling Pathway	2.217	6.07E-03	2	BMP10, SMAD4
