# Supplementary material for: Antimicrobial Peptides and Biomarkers Induced by Ultraviolet Irradiation Have the Potential to Reduce Endodontic Inflammation and Facilitate Tissue Healing
Source: Pharmaceutics. 2022 Sep 19;14(9):1979. doi: 10.3390/pharmaceutics14091979 (PMC9503046; doi:10.3390/pharmaceutics14091979)
Supplement: Supplementary file 1 [file pharmaceutics-14-01979-s001.zip › pharmaceutics-1897757-supplementary/Supplementary Table S4.pdf]

Supplementary Table S4. Antimicrobial peptides and biomarkers in the literature dataset (n=32) participating in the activation of innate and immune mechanisms as determined using IPA Diseases & Function annotations. Ingenuity Pathway Analysis (IPA, Qiagen, Germantown, MD) was used to assess whether the antimicrobial peptides and biomarkers in the literature dataset (n=32) would participate in the activation of innate and immune mechanisms. IPA Core Analysis was run on the list of 32 biomarkers and IPA Diseases & Function annotations were associated with cellular stress and injury (e.g., wound healing), cytokine signaling, growth factor signaling, intracellular and second messenger signaling, cellular growth, proliferation, and development, cellular immune response, and organismal growth and development.

Diseases & Functions Annotation	p-value	No. of molecules	Identification of molecules
**Antimicrobial Response, Inflammatory Response**			
Antibacterial response	1.66E-22	13	CAMP, CCL20, DEFB1, DEFB103A/DEFB103B, DEFB4A/DEFB4B, IL10, IL6, RNASE7, S100A12, S100A7, S100A8, S100A9, TNF
Antimicrobial response	6.55E-19	14	CAMP, CCL20, DEFB1, DEFB103A/DEFB103B, DEFB4A/DEFB4B, IL10, IL6, RNASE7, S100A12, S100A7, S100A8, S100A9, TGFB1, TNF
**Antimicrobial Response, Inflammatory Response**			
Chemoattraction	5.09E-23	12	CAMP, CCL2, CCL20, CSF2, CXCL1, CXCL3, CXCL8, DEFB4A/DEFB4B, FN1, TGFB1, TNF, VEGFA
Chemoattraction of leukocytes	5.45E-21	10	CAMP, CCL2, CCL20, CSF2, CXCL1, CXCL3, CXCL8, DEFB4A/DEFB4B, TGFB1, TNF
Chemoattraction of phagocytes	3.74E-17	8	CAMP, CCL2, CCL20, CXCL1, CXCL3, CXCL8, TGFB1, TNF
Chemoattraction of granulocytes	3.40E-16	7	CAMP, CCL2, CXCL1, CXCL3, CXCL8, TGFB1, TNF
Chemoattraction of neutrophils	2.60E-14	6	CAMP, CXCL1, CXCL3, CXCL8, TGFB1, TNF
**Cell Death and Survival**			
Apoptosis of neutrophils	5.50E-19	11	CAMP, CCL2, CSF2, CXCL1, CXCL2, CXCL8, DEFB103A/DEFB103B, IL10, IL6, TGFB1, TNF
Cell viability of blood cells	1.42E-16	13	CSF2, CXCL8, FGF2, FN1, ICAM1, IL10, IL6, SELE, SMAD3, SMAD4, TGFB1, TNF, VEGFA
Cell rolling of leukocytes	4.44E-16	9	CCL2, CXCL1, CXCL2, CXCL3, CXCL8, ICAM1, SELE, TNF, VEGFA
Cell death of immune cells	1.07E-15	17	CAMP, CCL2, CSF2, CXCL1, CXCL2, CXCL8, DEFB103A/DEFB103B, FN1, ICAM1, IL10, IL6, S100A8, SMAD3, SMAD4, TGFB1, TNF, VEGFA

Apoptosis of phagocytes	1.51E-15	12	CAMP, CCL2, CSF2, CXCL1, CXCL2, CXCL8, DEFB103A/DEFB103B, IL10, IL6, S100A8, TGFB1, TNF
Apoptosis of myeloid cells	1.95E-15	12	CAMP, CCL2, CSF2, CXCL1, CXCL2, CXCL8, DEFB103A/DEFB103B, IL10, IL6, S100A8, TGFB1, TNF
Apoptosis of blood cells	3.85E-15	15	CAMP, CCL2, CSF2, CXCL1, CXCL2, CXCL8, DEFB103A/DEFB103B, IL10, IL6, S100A8, SMAD3, SMAD4, TGFB1, TNF, VEGFA
Cell rolling of phagocytes	1.28E-14	7	CCL2, CXCL1, CXCL2, CXCL3, ICAM1, SELE, TNF
Cell viability of myeloid cells	2.25E-14	9	CSF2, CXCL8, FGF2, FN1, ICAM1, IL10, IL6, TGFB1, TNF
Apoptosis of leukocytes	3.69E-14	14	CAMP, CCL2, CSF2, CXCL1, CXCL2, CXCL8, DEFB103A/DEFB103B, IL10, IL6, S100A8, SMAD3, TGFB1, TNF, VEGFA
Cell rolling of granulocytes	3.83E-14	7	CXCL1, CXCL2, CXCL3, CXCL8, ICAM1, SELE, TNF
Cell death of hepatocytes	1.50E-06	6	CXCL3, IL10, IL6, SMAD3, TGFB1, TNF
Pathway	Pathway	Pathway	Pathway
**Cell-To-Cell Signaling and Interaction, Cellular Growth and Proliferation, Hematological System Development and Function**			
Stimulation of blood cells	1.93E-19	13	CAMP, CCL2, CCL20, CSF2, CXCL3, CXCL8, FGF2, FN1, IL10, IL6, SMAD3, TGFB1, TNF
Stimulation of myeloid cells	1.68E-18	10	CAMP, CCL20, CSF2, CXCL3, CXCL8, FN1, IL10, IL6, TGFB1, TNF
Stimulation of leukocytes	1.16E-17	12	CAMP, CCL2, CCL20, CSF2, CXCL3, CXCL8, FN1, IL10, IL6, SMAD3, TGFB1, TNF
Stimulation of connective tissue cells	5.55E-14	7	CSF2, FGF2, IL10, IL6, TGFB1, TNF, VEGFA
**Cell-To-Cell Signaling and Interaction, Cellular Movement, Hematological System Development and Function, Immune Cell Trafficking, Inflammatory Response**			
Cell movement of monocytes	1.37E-31	19	CAMP, CCL2, CCL20, CSF2, CXCL3, CXCL8, DEFB1, DEFB103A/DEFB103B, FN1, ICAM1, IL10, IL6, S100A12, S100A7, SELE, SMAD3, TGFB1, TNF, VEGFA
Cell movement of phagocytes	1.04E-29	25	CAMP, CCL2, CCL20, CSF2, CXCL1, CXCL2, CXCL3, CXCL8, DEFB1, DEFB103A/DEFB103B, DEFB4A/DEFB4B, FN1, ICAM1, IL10, IL6, S100A12, S100A7, S100A8, S100A9, SELE, SMAD3, SMAD4, TGFB1, TNF, VEGFA
Cell movement of myeloid cells	1.13E-29	25	CAMP, CCL2, CCL20, CSF2, CXCL1, CXCL2, CXCL3, CXCL8, DEFB1, DEFB103A/DEFB103B,

			DEFB4A/DEFB4B, FN1, ICAM1, IL10, IL6, S100A12, S100A7, S100A8, S100A9, SELE, SMAD3, SMAD4, TGFB1, TNF, VEGFA
Cell movement of neutrophils	2.01E-28	21	CAMP, CCL2, CSF2, CXCL1, CXCL2, CXCL3, CXCL8, DEFB1, DEFB103A/DEFB103B, DEFB4A/DEFB4B, FN1, ICAM1, IL10, IL6, S100A12, S100A8, S100A9, SELE, SMAD3, TGFB1, TNF
Cell movement of granulocytes	2.05E-28	22	CAMP, CCL2, CSF2, CXCL1, CXCL2, CXCL3, CXCL8, DEFB1, DEFB103A/DEFB103B, DEFB4A/DEFB4B, FN1, ICAM1, IL10, IL6, S100A12, S100A7, S100A8, S100A9, SELE, SMAD3, TGFB1, TNF
Cell movement of macrophages	1.21E-25	19	CAMP, CCL2, CSF2, CXCL1, CXCL3, CXCL8, DEFB1, DEFB103A/DEFB103B, DEFB4A/DEFB4B, FN1, ICAM1, IL10, IL6, S100A8, SMAD3, SMAD4, TGFB1, TNF, VEGFA
Cell movement of mononuclear leukocytes	3.41E-25	21	CAMP, CCL2, CCL20, CSF2, CXCL1, CXCL3, CXCL8, DEFB1, DEFB103A/DEFB103B, DEFB4A/DEFB4B, FN1, ICAM1, IL10, IL6, S100A12, S100A7, SELE, SMAD3, TGFB1, TNF, VEGFA
Cell movement of tumor cell lines	2.23E-24	25	CAMP, CCL2, CCL20, CSF2, CXCL1, CXCL2, CXCL3, CXCL8, DEFB103A/DEFB103B, DEFB4A/DEFB4B, FGF1, FGF2, FN1, IL6, PIGF, S100A7, S100A8, S100A9, SELE, SMAD3, SMAD4, TGFA, TGFB1, TNF, VEGFA
Cell movement of T lymphocytes	1.13E-21	16	CAMP, CCL2, CCL20, CXCL1, CXCL8, DEFB1, DEFB103A/DEFB103B, DEFB4A/DEFB4B, FN1, ICAM1, IL10, IL6, S100A7, SELE, TGFB1, TNF
Cell movement of colorectal cancer cell lines	1.03E-19	13	CAMP, CCL20, CXCL8, DEFB4A/DEFB4B, FGF2, IL6, S100A8, S100A9, SELE, SMAD4, TGFB1, TNF, VEGFA
Cell movement of carcinoma cell lines	4.63E-19	15	CAMP, CCL2, CSF2, CXCL3, CXCL8, FGF1, FGF2, FN1, IL6, PIGF, SELE, SMAD3, TGFA, TGFB1, TNF
Cell movement of leukocyte cell lines	6.77E-19	11	CCL2, CSF2, CXCL3, CXCL8, DEFB1, DEFB103A/DEFB103B, DEFB4A/DEFB4B, FN1, ICAM1, TNF, VEGFA
Cell movement of dendritic cells	2.20E-17	12	CCL2, CCL20, CSF2, CXCL8, DEFB1, DEFB103A/DEFB103B, DEFB4A/DEFB4B, ICAM1, IL10, SELE, TGFB1, TNF
Cell movement of mast cells	9.53E-17	9	CAMP, CCL2, CXCL8, DEFB1, DEFB103A/DEFB103B, DEFB4A/DEFB4B, ICAM1, SELE, TGFB1

Cell movement of leukemia cell lines	2.35E-16	11	CCL2, CCL20, CSF2, CXCL1, CXCL8, DEFB103A/DEFB103B, FN1, SELE, TGFB1, TNF, VEGFA
Cell movement of epithelial cells	2.65E-16	11	CXCL1, CXCL2, CXCL8, FGF2, FN1, ICAM1, IL6, SMAD3, TGFA, TGFB1, TNF
Cell movement of endothelial cells	2.88E-16	14	CAMP, CCL2, CSF2, CXCL1, CXCL8, FGF1, FGF2, FN1, ICAM1, SELE, SMAD3, TGFB1, TNF, VEGFA
Cell movement of kidney cell lines	3.25E-15	10	CAMP, CCL20, CXCL1, CXCL8, FGF2, FN1, ICAM1, SELE, TGFB1, VEGFA
Cell movement of cancer cells	3.54E-15	10	CCL2, CSF2, CXCL8, FN1, IL6, SELE, TGFA, TGFB1, TNF, VEGFA
Cell movement of embryonic cell lines	3.64E-14	10	CAMP, CCL20, CXCL1, FGF2, FN1, ICAM1, IL6, SELE, TGFB1, TNF
Cell movement of natural killer cells	3.81E-14	8	CCL2, CXCL3, CXCL8, ICAM1, IL10, SELE, TGFB1, TNF
Cell movement of endothelial cell lines	4.44E-14	9	BMP10, CXCL1, CXCL8, FGF2, FN1, SELE, TGFB1, TNF, VEGFA
Cell movement of hematopoietic cells	7.17E-14	8	CCL2, CCL20, CSF2, CXCL8, DEFB103A/DEFB103B, ICAM1, SELE, TNF
**Cell-To-Cell Signaling and Interaction, Cellular Movement, Hematological System Development and Function, Immune Cell Trafficking, Inflammatory Response**			
Recruitment of cells	7.85E-29	21	BMP10, CAMP, CCL2, CCL20, CSF2, CXCL1, CXCL2, CXCL3, CXCL8, DEFB4A/DEFB4B, FGF2, FN1, ICAM1, IL10, IL6, S100A8, SELE, SMAD3, TGFB1, TNF, VEGFA
Recruitment of blood cells	1.69E-27	20	BMP10, CAMP, CCL2, CCL20, CSF2, CXCL1, CXCL2, CXCL3, CXCL8, DEFB4A/DEFB4B, FN1, ICAM1, IL10, IL6, S100A8, SELE, SMAD3, TGFB1, TNF, VEGFA
Recruitment of phagocytes	4.77E-26	18	BMP10, CAMP, CCL2, CCL20, CSF2, CXCL1, CXCL2, CXCL3, CXCL8, DEFB4A/DEFB4B, ICAM1, IL10, IL6, S100A8, SELE, SMAD3, TGFB1, TNF
Recruitment of leukocytes	1.05E-25	19	BMP10, CAMP, CCL2, CCL20, CSF2, CXCL1, CXCL2, CXCL3, CXCL8, DEFB4A/DEFB4B, FN1, ICAM1, IL10, IL6, S100A8, SELE, SMAD3, TGFB1, TNF
Recruitment of myeloid cells	2.57E-25	18	BMP10, CAMP, CCL2, CCL20, CSF2, CXCL1, CXCL2, CXCL3, CXCL8, DEFB4A/DEFB4B, ICAM1, IL10, IL6, S100A8, SELE, SMAD3, TGFB1, TNF
Recruitment of neutrophils	1.37E-24	16	CAMP, CCL2, CCL20, CSF2, CXCL1, CXCL2, CXCL3, CXCL8, DEFB4A/DEFB4B, ICAM1, IL10, IL6, S100A8, SELE, SMAD3, TNF

Recruitment of mononuclear leukocytes	1.87E-23	14	BMP10, CAMP, CCL2, CCL20, CSF2, CXCL3, CXCL8, FN1, ICAM1, IL10, IL6, SELE, TGFB1, TNF
Recruitment of lymphatic system cells	3.36E-23	13	CCL2, CCL20, CSF2, CXCL3, CXCL8, FN1, ICAM1, IL10, IL6, SELE, TGFB1, TNF, VEGFA
Recruitment of lymphocytes	4.05E-21	12	CCL2, CCL20, CSF2, CXCL3, CXCL8, FN1, ICAM1, IL10, IL6, SELE, TGFB1, TNF
Recruitment of monocytes	4.84E-17	9	BMP10, CAMP, CCL2, CCL20, CSF2, CXCL3, CXCL8, ICAM1, TNF
Recruitment of T lymphocytes	7.02E-16	9	CCL2, CCL20, CSF2, CXCL3, CXCL8, ICAM1, IL10, SELE, TNF
**Cell-To-Cell Signaling and Interaction, Cellular Movement, Immune Cell Trafficking**			
Attraction of neutrophils	2.02E-16	7	CAMP, CCL2, CXCL1, CXCL3, CXCL8, TGFB1, TNF
Attraction of antigen presenting cells	1.14E-13	6	CAMP, CCL2, CCL20, CSF2, TGFB1, TNF
**Cell-To-Cell Signaling and Interaction, Hematological System Development and Function, Immune Cell Trafficking**			
Adhesion of phagocytes	3.76E-26	16	CCL2, CSF2, CXCL1, CXCL2, CXCL3, CXCL8, FN1, ICAM1, IL10, IL6, S100A8, S100A9, SELE, TGFB1, TNF, VEGFA
Adhesion of myeloid cells	1.59E-25	16	CCL2, CSF2, CXCL1, CXCL2, CXCL3, CXCL8, FN1, ICAM1, IL10, IL6, S100A8, S100A9, SELE, TGFB1, TNF, VEGFA
Adhesion of neutrophils	4.23E-25	14	CSF2, CXCL1, CXCL2, CXCL3, CXCL8, FN1, ICAM1, IL6, S100A8, S100A9, SELE, TGFB1, TNF, VEGFA
Adhesion of immune cells	5.19E-23	18	CAMP, CCL2, CCL20, CSF2, CXCL1, CXCL2, CXCL3, CXCL8, FN1, ICAM1, IL10, IL6, S100A8, S100A9, SELE, TGFB1, TNF, VEGFA
Adhesion of monocytes	3.54E-15	8	CCL2, CXCL8, FN1, ICAM1, IL10, SELE, TGFB1, TNF
Adhesion of mononuclear leukocytes	1.13E-14	10	CCL2, CCL20, CXCL8, FN1, ICAM1, IL10, IL6, SELE, TGFB1, TNF
Adhesion of endothelial cells	3.89E-14	10	CCL2, CXCL1, CXCL2, CXCL8, FN1, ICAM1, SELE, TGFB1, TNF, VEGFA
**Cell-To-Cell Signaling and Interaction, Hematological System Development and Function, Immune Cell Trafficking, Inflammatory Response**			
Activation of phagocytes	3.70E-27	21	CAMP, CCL2, CCL20, CSF2, CXCL1, CXCL3, CXCL8, DEFB4A/DEFB4B, FGF1, FN1, ICAM1, IL10, IL6, S100A12, S100A7, S100A8, S100A9, SELE, TGFB1, TNF, VEGFA

Activation of leukocytes	8.51E-27	24	CAMP, CCL2, CCL20, CSF2, CXCL1, CXCL3, CXCL8, DEFB4A/DEFB4B, FGF1, FGF2, FN1, ICAM1, IL10, IL6, S100A12, S100A7, S100A8, S100A9, SELE, SMAD3, SMAD4, TGFB1, TNF, VEGFA
Activation of cells	7.96E-26	26	CAMP, CCL2, CCL20, CSF2, CXCL1, CXCL2, CXCL3, CXCL8, DEFB4A/DEFB4B, FGF1, FGF2, FN1, ICAM1, IL10, IL6, S100A12, S100A7, S100A8, S100A9, SELE, SMAD3, SMAD4, TGFA, TGFB1, TNF, VEGFA
Activation of myeloid cells	2.83E-22	18	CAMP, CCL2, CSF2, CXCL1, CXCL3, CXCL8, FGF1, FN1, ICAM1, IL10, IL6, S100A12, S100A7, S100A8, S100A9, SELE, TGFB1, TNF
Activation of neutrophils	9.10E-21	12	CAMP, CCL2, CSF2, CXCL1, CXCL3, CXCL8, FN1, IL10, IL6, S100A7, SELE, TNF
Activation of antigen presenting cells	1.09E-19	16	CAMP, CCL2, CCL20, CSF2, CXCL8, DEFB4A/DEFB4B, FGF1, FN1, ICAM1, IL10, IL6, S100A9, SELE, TGFB1, TNF, VEGFA
Activation of mononuclear leukocytes	6.21E-19	17	CAMP, CCL2, CSF2, CXCL8, FGF1, FGF2, FN1, ICAM1, IL10, IL6, S100A12, S100A9, SMAD3, SMAD4, TGFB1, TNF, VEGFA
Activation of dendritic cells	2.62E-18	11	CAMP, CCL2, CCL20, CSF2, CXCL8, DEFB4A/DEFB4B, IL10, IL6, TGFB1, TNF, VEGFA
Activation of lymphocytes	1.07E-17	16	CAMP, CCL2, CSF2, CXCL8, FGF1, FGF2, FN1, ICAM1, IL10, IL6, S100A9, SMAD3, SMAD4, TGFB1, TNF, VEGFA
Activation of macrophages	3.80E-16	13	CAMP, CCL2, CSF2, CXCL8, FGF1, FN1, ICAM1, IL10, IL6, S100A9, SELE, TGFB1, TNF
Activation of T lymphocytes	1.27E-14	13	CCL2, CSF2, CXCL8, FGF1, FGF2, FN1, ICAM1, IL10, IL6, SMAD3, SMAD4, TGFB1, TNF
Activation of monocytes	4.73E-14	8	CAMP, CCL2, CSF2, CXCL8, FN1, IL10, S100A12, TNF
**Cell-To-Cell Signaling and Interaction, Hematological System Development and Function, Inflammatory Response**			
Binding of professional phagocytic cells	1.17E-29	19	CAMP, CCL2, CSF2, CXCL1, CXCL2, CXCL3, CXCL8, FGF1, FGF2, FN1, ICAM1, IL10, IL6, S100A8, S100A9, SELE, TGFB1, TNF, VEGFA
Binding of myeloid cells	3.52E-29	19	CAMP, CCL2, CSF2, CXCL1, CXCL2, CXCL3, CXCL8, FGF1, FGF2, FN1, ICAM1, IL10, IL6, S100A8, S100A9, SELE, TGFB1, TNF, VEGFA
Binding of leukocytes	6.08E-26	20	CAMP, CCL2, CCL20, CSF2, CXCL1, CXCL2, CXCL3, CXCL8, FGF1, FGF2, FN1, ICAM1, IL10, IL6, S100A8, S100A9, SELE, TGFB1, TNF, VEGFA

Binding of monocytes	2.21E-18	10	CCL2, CXCL8, FGF1, FGF2, FN1, ICAM1, IL10, SELE, TGFB1, TNF
Binding of endothelial cells	3.59E-18	13	CCL2, CXCL1, CXCL2, CXCL8, FGF1, FGF2, FN1, ICAM1, IL6, SELE, TGFB1, TNF, VEGFA
Binding of mononuclear leukocytes	1.13E-16	12	CCL2, CCL20, CXCL8, FGF1, FGF2, FN1, ICAM1, IL10, IL6, SELE, TGFB1, TNF
Binding of tumor cell lines	1.51E-16	15	CAMP, CCL2, CXCL2, CXCL3, CXCL8, FGF2, FN1, ICAM1, IL6, SELE, SMAD3, SMAD4, TGFB1, TNF, VEGFA
Response of neutrophils	4.30E-15	9	CAMP, CSF2, CXCL1, CXCL3, CXCL8, FN1, ICAM1, IL10, TNF
Binding of lymphoma cell lines	4.85E-15	9	CCL2, CXCL2, CXCL3, FN1, ICAM1, IL6, TGFB1, TNF, VEGFA
Response of myeloid leukocytes	5.81E-15	10	CAMP, CSF2, CXCL1, CXCL3, CXCL8, FN1, ICAM1, IL10, S100A9, TNF
Binding of vascular endothelial cells	7.68E-15	10	CCL2, CXCL8, FGF1, FGF2, FN1, ICAM1, SELE, TGFB1, TNF, VEGFA
Response of mononuclear leukocytes	2.01E-14	11	CAMP, CCL2, CSF2, CXCL8, FN1, ICAM1, IL10, IL6, SMAD3, TGFB1, TNF
**Cell-To-Cell Signaling and Interaction, Inflammatory Response**			
Immune response of cells	2.70E-18	18	CAMP, CCL2, CCL20, CSF2, CXCL1, CXCL3, CXCL8, FN1, ICAM1, IL10, IL6, S100A12, S100A8, S100A9, SMAD3, TGFB1, TNF, VEGFA
Immune response of myeloid cells	2.85E-17	13	CAMP, CCL2, CSF2, CXCL1, CXCL3, CXCL8, FN1, ICAM1, IL10, IL6, S100A9, TGFB1, TNF
Immune response of phagocytes	8.23E-17	13	CAMP, CCL2, CSF2, CXCL1, CXCL3, CXCL8, FN1, ICAM1, IL10, IL6, S100A9, TGFB1, TNF
Immune response of leukocytes	1.77E-16	14	CAMP, CCL2, CSF2, CXCL1, CXCL3, CXCL8, FN1, ICAM1, IL10, IL6, S100A9, SMAD3, TGFB1, TNF
Immune response of T lymphocytes	3.67E-14	9	CAMP, CSF2, CXCL8, FN1, ICAM1, IL10, IL6, TGFB1, TNF
Immune response of lymphocytes	4.87E-14	9	CSF2, CXCL8, FN1, ICAM1, IL10, IL6, SMAD3, TGFB1, TNF
Induction of cells	5.10E-14	9	CSF2, CXCL8, FGF2, IL10, IL6, SMAD3, TGFB1, TNF, VEGFA
**Cellular Development, Cellular Growth and Proliferation**			
Proliferation of hematopoietic progenitor cells	4.08E-19	14	CCL2, CCL20, CSF2, CXCL2, CXCL3, CXCL8, FGF2, IL10, IL6, S100A9, SMAD4, TGFB1, TNF, VEGFA
Proliferation of progenitor cells	1.17E-18	15	CCL2, CCL20, CSF2, CXCL2, CXCL3, CXCL8, FGF2, FN1, IL10, IL6, S100A9, SMAD4, TGFB1, TNF, VEGFA

Proliferation of endothelial cells	1.20E-18	15	CAMP, CCL2, CSF2, CXCL1, CXCL8, FGF1, FGF2, FN1, IL10, IL6, S100A8, S100A9, TGFB1, TNF, VEGFA
Proliferation of cancer cells	1.45E-18	16	CSF2, CXCL1, CXCL3, CXCL8, FGF1, FGF2, IL10, IL6, S100A7, S100A9, SMAD3, SMAD4, TGFA, TGFB1, TNF, VEGFA
Proliferation of myeloid progenitor cells	4.72E-16	8	CCL2, CCL20, CSF2, CXCL3, CXCL8, FGF2, IL6, TNF
Proliferation of connective tissue cells	7.16E-16	16	CCL2, CSF2, CXCL3, CXCL8, FGF1, FGF2, FN1, IL10, IL6, S100A8, S100A9, SMAD3, SMAD4, TGFA, TGFB1, TNF
Proliferation of blood cells	8.84E-16	18	CAMP, CCL2, CCL20, CSF2, CXCL2, CXCL3, CXCL8, FGF2, FN1, ICAM1, IL10, IL6, S100A9, SMAD3, SMAD4, TGFB1, TNF, VEGFA
Proliferation of synovial cells	3.21E-15	8	CCL2, CSF2, FGF2, FN1, IL10, IL6, TGFB1, TNF
Proliferation of myeloid cells	6.24E-15	11	CCL2, CCL20, CSF2, CXCL2, CXCL3, CXCL8, FGF2, IL10, IL6, TGFB1, TNF
Proliferation of bone marrow cells	3.50E-14	9	CCL2, CCL20, CSF2, CXCL3, CXCL8, FGF2, IL6, TGFB1, TNF
Maturation of cells	5.94E-14	13	CAMP, CCL2, CCL20, CSF2, FGF2, ICAM1, IL10, IL6, SMAD3, SMAD4, TGFB1, TNF, VEGFA
Proliferation of lymphatic system cells	6.34E-14	16	CAMP, CCL2, CCL20, CSF2, CXCL3, CXCL8, FGF2, FN1, ICAM1, IL10, IL6, SMAD3, SMAD4, TGFB1, TNF, VEGFA
Proliferation of muscle cells	7.20E-14	13	BMP10, CCL2, CXCL8, FGF1, FGF2, FN1, IL10, IL6, SMAD3, SMAD4, TGFB1, TNF, VEGFA
Proliferation of dermal cells	8.12E-14	10	CXCL8, FGF2, FN1, IL10, IL6, S100A8, SMAD3, TGFA, TGFB1, TNF
Proliferation of immune cells	9.44E-14	16	CAMP, CCL2, CSF2, CXCL2, CXCL3, CXCL8, FN1, ICAM1, IL10, IL6, S100A9, SMAD3, SMAD4, TGFB1, TNF, VEGFA
Proliferation of liver cells	1.87E-11	9	CSF2, CXCL3, FGF1, IL6, SMAD3, TGFA, TGFB1, TNF, VEGFA
Proliferation of hepatocytes	6.73E-11	8	CXCL3, FGF1, IL6, SMAD3, TGFA, TGFB1, TNF, VEGFA
Proliferation of heart cells	1.03E-08	6	BMP10, FGF1, FGF2, IL6, TGFB1, TNF
Proliferation of hepatic stellate cells	1.88E-06	4	IL6, SMAD3, TGFB1, TNF
Proliferation of cholangiocytes	4.26E-06	3	FGF2, IL6, TGFB1
Proliferation of cardiac fibroblasts	5.37E-06	3	FGF2, TGFB1, TNF
Proliferation of cardiomyocytes	6.45E-06	4	BMP10, FGF1, FGF2, IL6

Proliferation of mesangial cells	5.76E-05	3	IL6, TGFB1, TNF
Proliferation of tubular cells	3.24E-04	2	TGFB1, TNF
**Cellular Development, Cellular Growth and Proliferation, Hematological System Development and Function, Hematopoiesis, Lymphoid Tissue Structure and Development, Tissue Development**			
Development of endothelial tissue	2.99E-21	17	CAMP, CCL2, CSF2, CXCL1, CXCL2, CXCL8, FGF1, FGF2, FN1, IL10, IL6, PIGF, S100A8, S100A9, TGFB1, TNF, VEGFA
Development of antigen presenting cells	1.67E-14	11	CAMP, CCL2, CSF2, CXCL3, FN1, IL10, IL6, S100A9, TGFB1, TNF, VEGFA
**Cellular Growth and Proliferation**			
Angiogenesis	1.74E-23	24	BMP10, CAMP, CCL2, CSF2, CXCL1, CXCL2, CXCL8, FGF1, FGF2, FN1, ICAM1, IL10, IL6, PIGF, S100A12, S100A8, S100A9, SELE, SMAD3, SMAD4, TGFA, TGFB1, TNF, VEGFA
Proliferation of vascular cells	3.32E-20	16	CAMP, CCL2, CXCL1, CXCL8, FGF1, FGF2, FN1, IL10, IL6, S100A8, S100A9, SMAD3, SMAD4, TGFB1, TNF, VEGFA
Cell proliferation of vascular endothelial cells	1.21E-18	13	CAMP, CCL2, CXCL1, CXCL8, FGF1, FGF2, FN1, IL6, S100A8, S100A9, TGFB1, TNF, VEGFA
Formation of vessel	4.90E-14	10	CCL2, CSF2, FGF1, FGF2, FN1, ICAM1, IL6, SELE, TGFB1, VEGFA
**Cellular Movement, Hematological System Development and Function, Immune Cell Trafficking**			
Cell survival	1.73E-17	24	CAMP, CCL2, CSF2, CXCL1, CXCL2, CXCL3, CXCL8, DEFB103A/DEFB103B, DEFB4A/DEFB4B, FGF1, FGF2, FN1, ICAM1, IL10, IL6, S100A8, S100A9, SELE, SMAD3, SMAD4, TGFA, TGFB1, TNF, VEGFA
Cell viability	2.74E-15	22	CAMP, CCL2, CSF2, CXCL1, CXCL2, CXCL3, CXCL8, FGF1, FGF2, FN1, ICAM1, IL10, IL6, S100A8, S100A9, SELE, SMAD3, SMAD4, TGFA, TGFB1, TNF, VEGFA
**Cellular Movement, Hematological System Development and Function, Immune Cell Trafficking, Inflammatory Response**			
Chemotaxis	4.28E-37	27	CAMP, CCL2, CCL20, CSF2, CXCL1, CXCL2, CXCL3, CXCL8, DEFB1, DEFB103A/DEFB103B, DEFB4A/DEFB4B, FGF2, FN1, ICAM1, IL10, IL6, S100A12, S100A7, S100A8, S100A9, SELE, SMAD3, SMAD4, TGFA, TGFB1, TNF, VEGFA
Chemotaxis of leukocytes	1.43E-35	24	CAMP, CCL2, CCL20, CSF2, CXCL1, CXCL2, CXCL3, CXCL8, DEFB1, DEFB103A/DEFB103B,

			DEFB4A/DEFB4B, FN1, ICAM1, IL10, IL6, S100A12, S100A7, S100A8, S100A9, SELE, SMAD3, TGFB1, TNF, VEGFA
Chemotaxis of phagocytes	1.14E-33	22	CAMP, CCL2, CCL20, CSF2, CXCL1, CXCL2, CXCL3, CXCL8, DEFB1, DEFB103A/DEFB103B, DEFB4A/DEFB4B, ICAM1, IL10, S100A12, S100A7, S100A8, S100A9, SELE, SMAD3, TGFB1, TNF, VEGFA
Chemotaxis of myeloid cells	1.24E-33	22	CAMP, CCL2, CCL20, CSF2, CXCL1, CXCL2, CXCL3, CXCL8, DEFB1, DEFB103A/DEFB103B, DEFB4A/DEFB4B, ICAM1, IL10, S100A12, S100A7, S100A8, S100A9, SELE, SMAD3, TGFB1, TNF, VEGFA
Chemotaxis of granulocytes	2.41E-33	20	CAMP, CCL2, CSF2, CXCL1, CXCL2, CXCL3, CXCL8, DEFB1, DEFB103A/DEFB103B, DEFB4A/DEFB4B, ICAM1, IL10, S100A12, S100A7, S100A8, S100A9, SELE, SMAD3, TGFB1, TNF
Chemotaxis of neutrophils	1.17E-30	18	CAMP, CCL2, CSF2, CXCL1, CXCL2, CXCL3, CXCL8, DEFB1, DEFB103A/DEFB103B, DEFB4A/DEFB4B, ICAM1, S100A12, S100A8, S100A9, SELE, SMAD3, TGFB1, TNF
Chemotaxis of monocytes	1.26E-30	16	CAMP, CCL2, CCL20, CSF2, CXCL3, CXCL8, DEFB1, DEFB103A/DEFB103B, IL10, S100A12, S100A7, SELE, SMAD3, TGFB1, TNF, VEGFA
Migration of phagocytes	3.76E-30	21	CAMP, CCL2, CCL20, CSF2, CXCL1, CXCL2, CXCL3, CXCL8, DEFB1, DEFB103A/DEFB103B, DEFB4A/DEFB4B, FN1, ICAM1, IL10, IL6, S100A8, S100A9, SELE, TGFB1, TNF, VEGFA
Chemotaxis of mononuclear leukocytes	7.28E-30	18	CAMP, CCL2, CCL20, CSF2, CXCL3, CXCL8, DEFB1, DEFB103A/DEFB103B, DEFB4A/DEFB4B, FN1, IL10, S100A12, S100A7, SELE, SMAD3, TGFB1, TNF, VEGFA
Cell movement of antigen presenting cells	3.70E-27	21	CAMP, CCL2, CCL20, CSF2, CXCL1, CXCL3, CXCL8, DEFB1, DEFB103A/DEFB103B, DEFB4A/DEFB4B, FN1, ICAM1, IL10, IL6, S100A8, SELE, SMAD3, SMAD4, TGFB1, TNF, VEGFA
Migration of antigen presenting cells	7.22E-25	16	CCL2, CCL20, CSF2, CXCL1, CXCL3, CXCL8, DEFB1, DEFB103A/DEFB103B, DEFB4A/DEFB4B, FN1, ICAM1, IL10, S100A8, SELE, TNF, VEGFA
Migration of cells	1.06E-24	30	BMP10, CAMP, CCL2, CCL20, CSF2, CXCL1, CXCL2, CXCL3, CXCL8, DEFB1, DEFB103A/DEFB103B, DEFB4A/DEFB4B, FGF1, FGF2, FN1, ICAM1, IL10, IL6, PIGF, S100A12,

			S100A7, S100A8, S100A9, SELE, SMAD3, SMAD4, TGFA, TGFB1, TNF, VEGFA
Migration of mononuclear leukocytes	1.47E-23	19	CAMP, CCL2, CCL20, CSF2, CXCL1, CXCL3, CXCL8, DEFB1, DEFB103A/DEFB103B, DEFB4A/DEFB4B, FN1, ICAM1, IL10, IL6, S100A7, SELE, TGFB1, TNF, VEGFA
Migration of granulocytes	3.19E-23	15	CAMP, CCL2, CSF2, CXCL1, CXCL2, CXCL3, CXCL8, FN1, ICAM1, IL6, S100A8, S100A9, SELE, TGFB1, TNF
Migration of neutrophils	1.35E-22	14	CAMP, CSF2, CXCL1, CXCL2, CXCL3, CXCL8, FN1, ICAM1, IL6, S100A8, S100A9, SELE, TGFB1, TNF
Transmigration of phagocytes	6.34E-21	12	CCL2, CSF2, CXCL1, CXCL2, CXCL3, CXCL8, FN1, ICAM1, IL6, SELE, TGFB1, TNF
Cellular homeostasis	7.61E-21	26	CAMP, CCL2, CCL20, CSF2, CXCL1, CXCL2, CXCL3, CXCL8, DEFB1, DEFB103A/DEFB103B, DEFB4A/DEFB4B, FGF1, FGF2, FN1, ICAM1, IL10, IL6, PIGF, S100A8, S100A9, SMAD3, SMAD4, TGFA, TGFB1, TNF, VEGFA
Migration of monocytes	8.47E-21	12	CCL2, CCL20, CSF2, CXCL8, FN1, ICAM1, IL10, IL6, SELE, TGFB1, TNF, VEGFA
Migration of macrophages	9.77E-21	12	CCL2, CSF2, CXCL1, CXCL3, CXCL8, DEFB1, DEFB103A/DEFB103B, DEFB4A/DEFB4B, FN1, S100A8, TNF, VEGFA
Transmigration of leukocytes	1.96E-20	13	CCL2, CSF2, CXCL1, CXCL2, CXCL3, CXCL8, FN1, ICAM1, IL6, SELE, TGFB1, TNF, VEGFA
Chemotaxis of lymphocytes	5.40E-20	12	CAMP, CCL2, CCL20, CXCL3, CXCL8, DEFB1, DEFB103A/DEFB103B, DEFB4A/DEFB4B, FN1, IL10, S100A7, TGFB1
Chemotaxis of T lymphocytes	1.44E-19	11	CAMP, CCL2, CCL20, CXCL8, DEFB1, DEFB103A/DEFB103B, DEFB4A/DEFB4B, FN1, IL10, S100A7, TGFB1
Chemotaxis of antigen presenting cells	9.50E-19	12	CAMP, CCL2, CCL20, CSF2, DEFB1, DEFB103A/DEFB103B, DEFB4A/DEFB4B, ICAM1, IL10, SMAD3, TGFB1, TNF
Cellular infiltration	1.16E-18	18	CCL2, CSF2, CXCL2, CXCL3, CXCL8, FGF2, FN1, ICAM1, IL10, IL6, S100A8, S100A9, SELE, SMAD3, SMAD4, TGFB1, TNF, VEGFA
Mobilization of cells	4.07E-18	10	CCL2, CCL20, CSF2, CXCL2, CXCL3, CXCL8, FGF2, SELE, TNF, VEGFA
Cellular infiltration by phagocytes	1.36E-17	15	CCL2, CSF2, CXCL2, CXCL3, CXCL8, ICAM1, IL10, IL6, S100A8, S100A9, SELE, SMAD3, SMAD4, TGFB1, TNF

Cellular infiltration by myeloid cells	5.20E-17	15	CCL2, CSF2, CXCL2, CXCL3, CXCL8, ICAM1, IL10, IL6, S100A8, S100A9, SELE, SMAD3, SMAD4, TGFB1, TNF
Migration of leukocyte cell lines	5.91E-17	9	CCL2, CXCL3, CXCL8, DEFB1, DEFB103A/DEFB103B, DEFB4A/DEFB4B, ICAM1, TNF, VEGFA
Mobilization of hematopoietic progenitor cells	6.23E-17	8	CCL2, CCL20, CSF2, CXCL2, CXCL3, CXCL8, SELE, VEGFA
Migration of vascular cells	8.23E-17	13	CCL2, CSF2, CXCL1, CXCL8, FGF1, FGF2, FN1, IL6, SELE, SMAD3, TGFB1, TNF, VEGFA
Cellular infiltration by leukocytes	2.18E-16	16	CCL2, CSF2, CXCL2, CXCL3, CXCL8, FN1, ICAM1, IL10, IL6, S100A8, S100A9, SELE, SMAD3, SMAD4, TGFB1, TNF
Migration of keratinocytes	1.09E-15	9	CXCL1, CXCL8, FN1, ICAM1, IL6, SMAD3, TGFA, TGFB1, TNF
Migration of epithelial cells	2.50E-15	10	CXCL1, CXCL2, CXCL8, FN1, ICAM1, IL6, SMAD3, TGFA, TGFB1, TNF
Migration of endothelial cells	3.01E-15	13	CCL2, CSF2, CXCL1, CXCL8, FGF1, FGF2, FN1, ICAM1, SELE, SMAD3, TGFB1, TNF, VEGFA
Transmigration of myeloid cells	3.36E-15	9	CCL2, CXCL1, CXCL2, CXCL3, CXCL8, FN1, ICAM1, IL6, TNF
Mobilization of phagocytes	3.47E-15	7	CCL2, CCL20, CSF2, CXCL2, CXCL3, CXCL8, TNF
Migration of tumor cells	6.81E-15	11	CCL2, CSF2, CXCL1, CXCL8, FN1, IL6, SELE, TGFA, TGFB1, TNF, VEGFA
Mobilization of neutrophils	1.44E-14	6	CCL2, CSF2, CXCL2, CXCL3, CXCL8, TNF
Transmigration of neutrophils	2.42E-14	8	CXCL1, CXCL2, CXCL3, CXCL8, FN1, ICAM1, IL6, TNF
TH1 immune response of T lymphocytes	2.60E-14	6	CXCL8, ICAM1, IL10, IL6, TGFB1, TNF
Transendothelial migration of neutrophils	5.55E-14	7	CXCL1, CXCL2, CXCL8, FN1, ICAM1, IL6, TNF
Chemotaxis of dendritic cells	6.70E-14	8	CCL2, CCL20, DEFB1, DEFB103A/DEFB103B, DEFB4A/DEFB4B, IL10, TGFB1, TNF
**Cellular Movement, Immune Cell Trafficking**			
Leukocyte migration	1.71E-26	26	BMP10, CAMP, CCL2, CCL20, CSF2, CXCL1, CXCL2, CXCL3, CXCL8, DEFB1, DEFB103A/DEFB103B, DEFB4A/DEFB4B, FN1, ICAM1, IL10, IL6, S100A12, S100A7, S100A8, S100A9, SELE, SMAD3, SMAD4, TGFB1, TNF, VEGFA
**Hematological System Development and Function, Immune Cell Trafficking, Inflammatory Response, Tissue Development**			

Accumulation of leukocytes	3.18E-23	17	CCL2, CSF2, CXCL1, CXCL2, CXCL3, CXCL8, FGF1, FGF2, ICAM1, IL10, IL6, S100A8, S100A9, SELE, TGFB1, TNF, VEGFA
Accumulation of phagocytes	2.49E-21	14	CCL2, CSF2, CXCL1, CXCL2, CXCL3, CXCL8, FGF1, FGF2, IL10, IL6, S100A8, S100A9, TNF, VEGFA
Accumulation of myeloid cells	3.19E-20	14	CCL2, CSF2, CXCL1, CXCL2, CXCL3, CXCL8, FGF1, FGF2, IL10, IL6, S100A8, S100A9, TNF, VEGFA
Accumulation of neutrophils	2.47E-19	11	CCL2, CSF2, CXCL1, CXCL2, CXCL3, CXCL8, IL10, IL6, S100A8, S100A9, TNF
Accumulation of mononuclear leukocytes	5.15E-15	10	CCL2, CSF2, CXCL2, CXCL3, ICAM1, IL6, SELE, TGFB1, TNF, VEGFA
**Hematological System Development and Function, Inflammatory Response, Tissue Morphology**			
Quantity of phagocytes	9.34E-23	19	CAMP, CCL2, CCL20, CSF2, CXCL2, CXCL8, FGF1, ICAM1, IL10, IL6, PI3, S100A8, S100A9, SELE, SMAD3, TGFA, TGFB1, TNF, VEGFA
Quantity of neutrophils	1.16E-22	16	CAMP, CCL2, CSF2, CXCL2, FGF1, ICAM1, IL10, IL6, PI3, S100A8, S100A9, SELE, SMAD3, TGFA, TGFB1, TNF
Quantity of granulocytes	2.68E-22	17	CAMP, CCL2, CSF2, CXCL2, CXCL8, FGF1, ICAM1, IL10, IL6, PI3, S100A8, S100A9, SELE, SMAD3, TGFA, TGFB1, TNF
Quantity of myeloid cells	2.41E-19	18	CAMP, CCL2, CSF2, CXCL2, CXCL8, FGF1, ICAM1, IL10, IL6, PI3, S100A8, S100A9, SELE, SMAD3, TGFA, TGFB1, TNF, VEGFA
Quantity of leukocytes	5.63E-18	20	CAMP, CCL2, CCL20, CSF2, CXCL1, CXCL2, CXCL8, FGF1, ICAM1, IL10, IL6, PI3, S100A8, S100A9, SELE, SMAD3, TGFA, TGFB1, TNF, VEGFA
Quantity of cells	1.28E-15	23	CAMP, CCL2, CCL20, CSF2, CXCL1, CXCL2, CXCL8, FGF1, FGF2, FN1, ICAM1, IL10, IL6, PI3, S100A8, S100A9, SELE, SMAD3, SMAD4, TGFA, TGFB1, TNF, VEGFA
Quantity of dendritic cells	1.22E-13	9	CAMP, CCL2, CCL20, CSF2, IL10, PI3, TGFB1, TNF, VEGFA
**Inflammatory Response**			
Inflammatory response	9.34E-29	26	CAMP, CCL2, CCL20, CSF2, CXCL1, CXCL2, CXCL3, CXCL8, DEFB1, DEFB103A/DEFB103B, DEFB4A/DEFB4B, FGF1, FGF2, FN1, ICAM1, IL10, IL6, S100A12, S100A7, S100A8, S100A9, SELE, SMAD3, TGFB1, TNF, VEGFA
Invasion of cells	4.64E-21	23	CAMP, CCL2, CSF2, CXCL1, CXCL2, CXCL8, DEFB103A/DEFB103B, FGF1, FGF2, FN1, ICAM1,

			IL10, IL6, S100A12, S100A7, S100A8, S100A9, SMAD3, SMAD4, TGFA, TGFB1, TNF, VEGFA
Proinflammatory response	4.92E-15	7	CCL2, CXCL3, CXCL8, IL10, IL6, TNF, VEGFA
Innate immune response	5.94E-14	10	CAMP, CXCL1, CXCL8, FN1, IL10, IL6, RNASE7, S100A12, SMAD3, TNF
**Tissue Development**			
Healing of wound	1.53E-20	13	CSF2, FGF1, FGF2, FN1, ICAM1, IL10, IL6, SMAD3, SMAD4, TGFA, TGFB1, TNF, VEGFA
Development of epithelial tissue	1.69E-20	18	CAMP, CCL2, CSF2, CXCL1, CXCL2, CXCL8, FGF1, FGF2, FN1, IL10, IL6, PIGF, S100A8, S100A9, SMAD4, TGFB1, TNF, VEGFA
Wound	6.95E-20	14	CAMP, CSF2, FGF1, FGF2, FN1, ICAM1, IL10, IL6, SMAD3, SMAD4, TGFA, TGFB1, TNF, VEGFA
Growth of epithelial tissue	4.41E-19	19	CAMP, CCL2, CSF2, CXCL1, CXCL3, CXCL8, FGF1, FGF2, FN1, IL10, IL6, S100A8, S100A9, SMAD3, SMAD4, TGFA, TGFB1, TNF, VEGFA
Proliferation of epithelial cells	3.78E-17	16	CAMP, CSF2, CXCL3, CXCL8, FGF1, FGF2, FN1, IL10, IL6, S100A8, SMAD3, SMAD4, TGFA, TGFB1, TNF, VEGFA
Formation of skin	5.42E-16	13	CAMP, CSF2, FGF2, FN1, IL10, IL6, S100A7, S100A8, SMAD3, TGFA, TGFB1, TNF, VEGFA
Cell-mediated response of T lymphocytes	2.36E-15	8	CSF2, CXCL8, FN1, ICAM1, IL10, IL6, TGFB1, TNF
Cell proliferation of fibroblasts	5.66E-15	13	CCL2, CSF2, CXCL3, FGF1, FGF2, FN1, IL10, IL6, S100A9, SMAD3, TGFA, TGFB1, TNF
Cell movement of memory T lymphocytes	1.10E-14	7	CCL2, CCL20, DEFB1, DEFB103A/DEFB103B, DEFB4A/DEFB4B, ICAM1, TNF
